# Inhibition of the H3K9 methyltransferase G9A attenuates oncogenicity and activates the hypoxia signaling pathway

**DOI:** 10.1371/journal.pone.0188051

**Published:** 2017-11-16

**Authors:** Jolene Caifeng Ho, Lissa Nurrul Abdullah, Qing You Pang, Sudhakar Jha, Edward Kai-Hua Chow, Henry Yang, Hiroyuki Kato, Lorenz Poellinger, Jun Ueda, Kian Leong Lee

**Affiliations:** 1 Cancer Stem Cells and Biology Program, Cancer Science Institute of Singapore, National University of Singapore, Singapore, Singapore; 2 Department of Cell and Molecular Biology, Karolinska Institutet, Stockholm, Sweden; 3 Center for Advanced Research and Education, Asahikawa Medical University, Asahikawa, Hokkaido, Japan; University of South Alabama Mitchell Cancer Institute, UNITED STATES

## Abstract

Epigenetic mechanisms play important roles in the regulation of tumorigenesis, and hypoxia-induced epigenetic changes may be critical for the adaptation of cancer cells to the hypoxic microenvironment of solid tumors. Previously, we showed that loss-of-function of the hypoxia-regulated H3K9 methyltransferase G9A attenuates tumor growth. However, the mechanisms by which blockade of G9A leads to a tumor suppressive effect remain poorly understood. We show that G9A is highly expressed in breast cancer and is associated with poor patient prognosis, where it may function as a potent oncogenic driver. In agreement with this, G9A inhibition by the small molecule inhibitor, BIX-01294, leads to increased cell death and impaired cell migration, cell cycle and anchorage-independent growth. Interestingly, whole transcriptome analysis revealed that genes involved in diverse cancer cell functions become hypoxia-responsive upon G9A inhibition. This was accompanied by the upregulation of the hypoxia inducible factors HIF1α and HIF2α during BIX-01294 treatment even in normoxia that may facilitate the tumor suppressive effects of BIX-01294. HIF inhibition was able to reverse some of the transcriptional changes induced by BIX-01294 in hypoxia, indicating that the HIFs may be important drivers of these derepressed target genes. Therefore, we show that G9A is a key mediator of oncogenic processes in breast cancer cells and G9A inhibition by BIX-01294 can successfully attenuate oncogenicity even in hypoxia.

## Introduction

As solid tumors expand, they increase uptake of oxygen, leading to the heterogeneous formation of hypoxic areas within tumor cores. Cells classically respond to hypoxia by activating the hypoxia-inducible transcription factors HIF1α and HIF2α, which induce changes in the gene expression of cancer cells. Many of these hypoxia target genes are important for cellular adaptation to low oxygen conditions, driving the acquisition of additional hallmarks of oncogenesis to promote and support cancer progression [1]. Consequently, cancer cells adapted to hypoxia develop more malignant phenotypes and exhibit increased resistance to therapy [2,3].

Some of the target genes induced by hypoxia include epigenetic factors [4–6], indicating a link between hypoxia and epigenetic changes in cancer cells that may be key to their long-term adaptation. The hypoxia-regulated epigenetic factor G9A (also known as EHMT2), a histone H3 lysine 9 (H3K9) methyltransferase [7,8], has been implicated as an important oncogenic driver in multiple cancers [9–11]. Hypoxia has been shown to induce H3K9 dimethylation (H3K9me2) in a global and gene-specific manner through the upregulation of both G9A protein level and activity [4]. H3K9 methylation mediates gene silencing and heterochromatin formation [12–14], and aberrant H3K9me2 has been implicated in cancer [5]. Consistent with previous reports [9], we also found that G9A was upregulated in many types of cancers including breast and cervical cancer, leading to reduced patient survival. Therefore, H3K9 methylation mediated by G9A constitutes a key consideration in the study of long-term epigenetic adaptation in hypoxic cancer cells.

We previously demonstrated that loss-of-function of G9a leads to attenuation of tumor growth [6]. This was recapitulated using BIX-01294, a small molecule inhibitor of G9a, highlighting the therapeutic potential of targeted G9A treatment regimens in the control of tumorigenesis. BIX-01294 inhibits the methyltransferase activity of G9A and its heterodimer partner G9a-like protein (GLP) by blocking their active sites. This prevents interactions with the histone H3 tail for the attachment of the methyl group to H3K9 [15]. BIX-01294 has been shown to specifically block G9A and GLP function with minimal effects on other histone or protein methyltransferases such as SUV39H1 and PRMT1 [16]. Therefore, BIX-01294 and its analogues that are effective in blocking G9A/GLP are of particular interest in therapeutic strategies involving the inhibition of epigenetic factors for the treatment of cancer. However, to apply small molecule inhibitors of G9A in cancer therapeutic strategies, their roles in attenuating oncogenic function need to be clarified.

To elucidate how G9A inhibition suppresses tumorigenesis, G9A was pharmacologically inhibited in proof-of-concept experiments in the breast cancer cell line, MCF-7, and the cervical cancer cell line, HeLa. G9A inhibition in MCF-7 using BIX-01294 significantly reduced cell migration and colony forming potential, and increased cell cycle arrest and apoptosis. This was accompanied by decreased H3K9me2 both globally and in a gene-specific manner. Therefore, G9A inhibition was effective in curtailing a number of cancer functions examined.

Given that G9A is hypoxia-regulated [4,6], we further examined the role of the hypoxia pathway during G9A inhibition by BIX-01294. Unexpectedly, inhibition of G9A upregulated the HIFs even without physiological hypoxia as the classical stimulus. In parallel, G9A inhibition with the reduction of the H3K9me2 repressive mark also led to the derepression of previously silenced hypoxia target genes, many of which are involved in cell cycle and survival functions. Together, this suggests that cancer cells may be able to mount a survival response against drug-induced attenuation of cellular growth via the activation of the HIF pathway.

Our findings indicate that G9A inhibition by BIX-01294, while effective in reducing cancer cell growth, also unexpectedly activates the HIF pathway. Hypoxia is a hallmark of solid tumors and hypoxia-induced epigenetic changes, such as those mediated via G9A, may be critical in driving some of the adaptive changes in cancer cells and thus play essential roles in the regulation of tumorigenesis. BIX-01294 treatment in cancer cells may thus benefit from the simultaneous inhibition of the HIF pathway to minimize the survival response of cancer cells and prevent relapse, where even a small number of cells that escape eradication may return as refractory disease. Testing of this hypothesis revealed that while singular inhibition of G9A or the HIFs was effective in reducing cancer cell viability, co-treatment with both drugs resulted in a reduced effect, suggesting that the HIFs may in fact facilitate the therapeutic effects of G9A inhibition and HIF inhibition may be antagonistic to these effects.

The expression and activity of hypoxia-regulated G9A as well as its prominent role in cancer makes it an ideal candidate for the investigation of the role of epigenetics in hypoxic cancer cells. This study provides additional insights into the epigenetic mechanisms of cancer, and is important in the development of novel strategies for therapeutic intervention such as through G9A inhibition.

## Materials and methods

### Cell culture and treatments

Human breast cancer (MCF-7) and cervical cancer (HeLa) cell lines (ATCC, VA, USA) were maintained in DMEM (Nacalai Tesque, Kyoto, Japan) containing 10% FBS (Sigma-Aldrich, MO, USA), 100 U/ml penicillin, 100 μg/ml streptomycin, 2 mM GlutaMAX, 55 μM β-mercaptoethanol and 100 μM non-essential amino acids (Invitrogen, CA, USA). For acute hypoxia treatment, cells were cultured for 20 hours in normoxia (21% O_2_ and 5% CO_2_) followed by 4 hours in hypoxia (1% O_2_ [17] and 5% CO_2_) at 37°C in an Invivo_2_ Hypoxia Workstation 400 (Ruskinn Technology, Leeds, UK). Chronic hypoxia and normoxia treatments consist of 24 hours in hypoxia or normoxia respectively [18]. For BIX-01294 and acriflavine (ACF) treatments, cells were treated with media containing 6 μM BIX-01294 (Stemgent, MA, USA) and/or 0.6 μM ACF (Sigma-Aldrich) in 0.1% dimethyl sulfoxide (DMSO, Sigma-Aldrich) for 24 hours unless otherwise stated, with 0.1% DMSO in media as control.

### Protein extraction and western blot analyses

Mammalian Protein Extraction Reagent (M-PER, Thermo Scientific, IL, USA) was used to harvest total protein for western analyses except for HIF2α, which was harvested using the NE-PER Nuclear and Cytoplasmic Extraction Reagents (Thermo Scientific). Triton extraction buffer was used to harvest histones by acid extraction according to the Abcam histone extraction protocol. Protein concentrations were determined using the Bio-Rad Protein Assay Kit (Bio-Rad, CA, USA). For western blot, primary antibodies used were anti-G9A (Sigma-Aldrich, G6919, 1:1000), anti-JMJD1A (Abcam, Cambridge, UK, ab80598, 1:1000), anti-HIF1α and anti-HIF2α (Novus, CO, USA, NB100-479, 1:1000 and NB100-122, 1:1000 respectively), anti-β-actin (Santa Cruz Biotechnology, TX, USA, sc-1616, 1:500), anti-histone H3 (Millipore, Carrigtwohill, Ireland, 06–755, 1:100), and anti-H3K9me1/2/3 (Active Motif, CA, USA, MABI0306, 1:500, MABI0307, 1:1000 and MABI0308, 1:500 respectively).

### RT-qPCR analysis

Total RNA was extracted using the RNeasy Mini Kit (Qiagen, Düsseldorf, Germany), and cDNA synthesized using the iScript cDNA synthesis kit (Bio-Rad) following manufacturer protocols. RT-qPCR primer sequences are shown in S1 Table and gene expression was quantified on the BioMark real-time PCR system (Fluidigm, CA, USA) according to manufacturer specifications.

### Scratch wound healing assay

Cells were plated to confluency in 12-well plates and allowed to attach overnight. A sterile P10 pipette tip was drawn vertically down the center of each well to create the scratch wound. Cell media was changed and cells were subjected to treatments as indicated. Photographs were taken every hour for 32 hours after the creation of the scratch wound using the Digital Eclipse C1 Plus confocal microscope (Nikon, Tokyo, Japan). The area of the wound was calculated using the NIS-Elements Imaging Software (Nikon). The rate of cell migration was calculated using the difference between the wound area at each time point and the total initial wound area at 0h.

### Colony formation assay

2×10^3^ cells were seeded into a working layer of 0.35% low melt agar and overlaid on a base layer of 0.5% low melt agar, both in DMEM with 10% FBS, in 12-well plates. Cells were grown in normoxia or hypoxia with their respective treatments at 37°C for 21 days. Colonies were stained with gentian violet (Integrated Contract Manufacturing Pharma, Singapore) diluted 1:50 with PBS. The number of colonies was counted using ImageJ v1.48 [19].

### *G9A* shRNA knockdown

Knockdown of *G9A* expression was performed using lentivirally expressed shRNAs (Sigma-Aldrich) in MCF-7 cells. Recombinant viruses were prepared by co-transfection of shRNA vectors or empty vector as knockdown control with pLKO.1 packaging plasmids in HEK 293T cells. Viral supernatants were filtered through 0.45 μm Millex HA filters (Millipore) and infections were performed with 4 μg/ml polybrene (Sigma-Aldrich). Antibiotic selection was carried out with 2 μg/ml puromycin (Sigma-Aldrich). Sequences used for *G9A* knockdown are shown in S2 Table.

### Chromatin immunoprecipitation (ChIP)

ChIP was carried out using the Agilent Mammalian ChIP-on-chip protocol v9.1 to the ChIP DNA purification step, with genomic DNA sonicated to 300–600 bp fragments using the Bioruptor (Diagenode, Liège, Belgium) at 30 seconds on and 30 seconds off at the highest intensity. Antibodies used were anti-H3K9me2 (Active Motif, MABI0307), anti-H3K4me3 (Active Motif, MABI0304) and mouse IgG (Santa Cruz Biotechnology, sc-2025). Primers used for ChIP-qPCR analysis are shown in S3 Table. MCF-7 reference and ChIP-seq tracks used were from GSE57498 for ChromHMM [20], GSE35583 for H3K4me3 [21], GSE56826 for H3K9me2 [22], GSE67206 for G9A [23], and GSE28352 for HIF1α and HIF2α [24].

### Microarray analysis

Total RNA was harvested using the RNeasy Mini Kit and resolved on the 2100 Bioanalyzer (Agilent, CA, USA) for determination of RNA quality. High purity and integrity samples with 260/280 and 260/230 absorbance ratios > 1.8 and RNA integrity numbers (RIN) > 8.0 were reverse transcribed into cDNA and *in vitro* transcribed into biotin-labelled cRNA using the Illumina TotalPrep RNA Amplification kit (Illumina, CA, USA). This was hybridized on HumanHT-12 v4 BeadChips (Illumina) and scanned on the BeadArray Reader (Illumina) at scan factor 2. Raw intensity values were subjected to background subtraction on the BeadStudio Data Analysis Software v3.1.3.0 (Illumina) and normalized using the cross-correlation method [25]. Differential gene expression was identified based on a fold change cutoff of > 1.5 and t-test *P* value < 0.01 compared to the average of the untreated, DMSO or BIX-01294-treated normoxia controls. The microarray data was deposited into NCBI GEO with accession number GSE89891.

### Gene ontology analysis

Gene ontology analysis was carried out using Ingenuity Pathway Analysis (IPA, Qiagen) v21249400. Genes up- or downregulated by at least 1.5-fold with t-test *P* value < 0.01 were identified from the microarray results and used as input for IPA. Significant associations with functional categories were limited to experimentally observed results in human only, using Fisher's exact test at a cutoff threshold of *P* ≤ 0.05.

### Flow cytometry

For cell cycle analysis, cells were harvested and fixed in 70% ethanol overnight at -20°C before staining with propidium iodide (PI) DNA staining solution (10 μg/ml PI, 100 μg/ml RNase A and 0.1% Triton-X 100 in PBS). For apoptosis analysis, cells were washed with Annexin V binding buffer (BD Pharmingen, NJ, USA) and stained with Annexin V Alexa Fluor 647 conjugate and SYTOX Blue at 1:1000 each (Thermo Scientific) before analysis using the LSR II Flow Cytometry System (BD Pharmingen).

### Trypan blue exclusion assay

Cells were trypsinized and 10 μl of the cell solution was stained with trypan blue solution (HyClone Laboratories, UT, USA) in a 1:1 ratio. Using a hemocytometer, cells stained blue were counted as dead cells while cells that remained unstained were counted as viable cells. The number of dead cells is presented as a percentage over the total number of cells in each sample.

### Statistical analyses

Data are presented as means ± SEM (standard error of the mean). Statistical significance was calculated using Student’s t-test unless otherwise stated. *P* values less than 0.05 were considered statistically significant and indicated as *, *P* < 0.05; **, *P* < 0.01; ***, *P* < 0.001.

## Results

### *G9A* is overexpressed in multiple cancers and higher levels of G9A portend poorer prognosis

**Fig 1:**
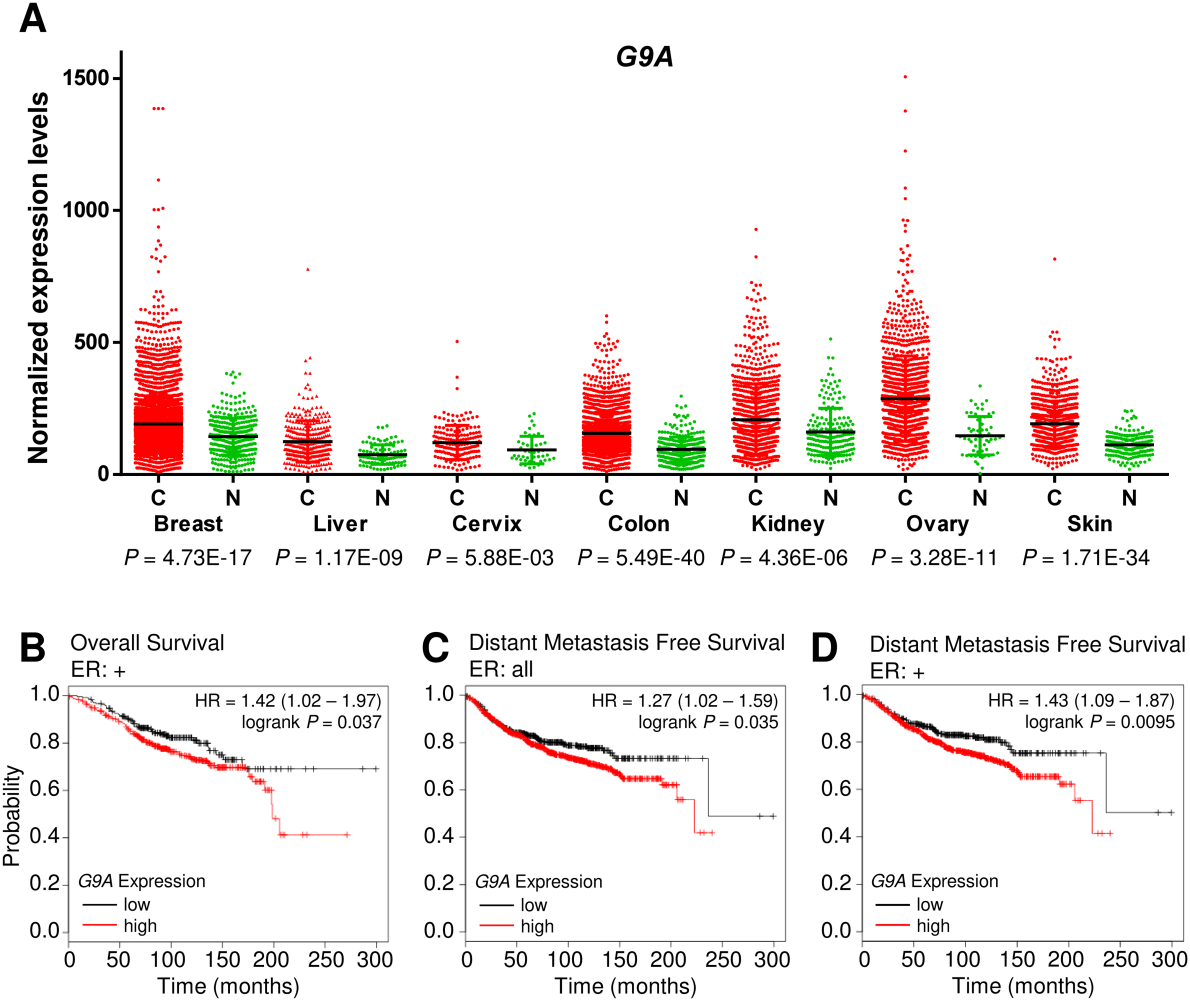
G9A is upregulated in diverse malignancies and is a predictor of poor survival in breast cancer. (A) Scatter plot showing increased *G9A* expression in cancers (C, red) compared to normal tissues (N, green) from the GENT database. Black lines indicate average gene expression levels. (B) Kaplan-Meier Plotter data showing the probability of overall survival in ER+ breast cancer patients and (C) distant metastasis free survival in all breast cancer patients and (D) ER+ breast cancer patients, with high *G9A* expression (red line) compared to low *G9A* expression (black line; HR, hazard ratio).

We previously showed that *G9a*-deficient cells form smaller tumors compared to wild-type controls and the use of the G9A inhibitor BIX-01294 reduced tumor formation, suggesting that G9A inhibition may have anti-tumorigenic effects [6]. To assess the utility of G9A inhibition as a cancer therapy, we identified cancers with *G9A* overexpression as these are most likely to respond favorably. Using the Gene Expression across Normal and Tumor tissue database (GENT, http://mgrc.kribb.re.kr/), we found that *G9A* expression was significantly upregulated in multiple cancers (Fig 1A). *G9A* upregulation in breast and cervical cancers was further confirmed using the Genevestigator database [26], which showed an increase in *G9A* expression in patient cancer tissues and the corresponding cancer cell lines compared to normal breast and cervical tissues respectively (S1 Fig). G9A upregulation was also reported by other studies in various cancers [9–11], suggesting that G9A may have a pan-cancer role in driving oncogenesis and its inhibition may have broad therapeutic applications.

In breast cancer, there was strong upregulation of *G9A* expression with high statistical significance. To determine the association of *G9A* overexpression in breast cancer patients with clinical outcome, *G9A* expression levels and the corresponding survival data were examined using the Kaplan-Meier Plotter [27] database of microarray gene expression. We found that *G9A* expression is negatively correlated with overall survival (OS) in estrogen receptor positive (ER+) breast cancer patients (Fig 1B). *G9A* expression was also associated with metastasis, where analysis of data on distant metastasis free survival (DMFS, Fig 1C) indicated that metastases were more likely to occur for breast cancer patients with high *G9A* expression. In ER+ breast cancer patients, DMFS was also significantly lowered when *G9A* expression was high (Fig 1D). These results indicate that high *G9A* levels are associated with tumorigenic development leading to poorer patient prognosis.

### BIX-01294 decreases H3K9 methylation and dysregulates the expression of G9A target genes

**Fig 2:**
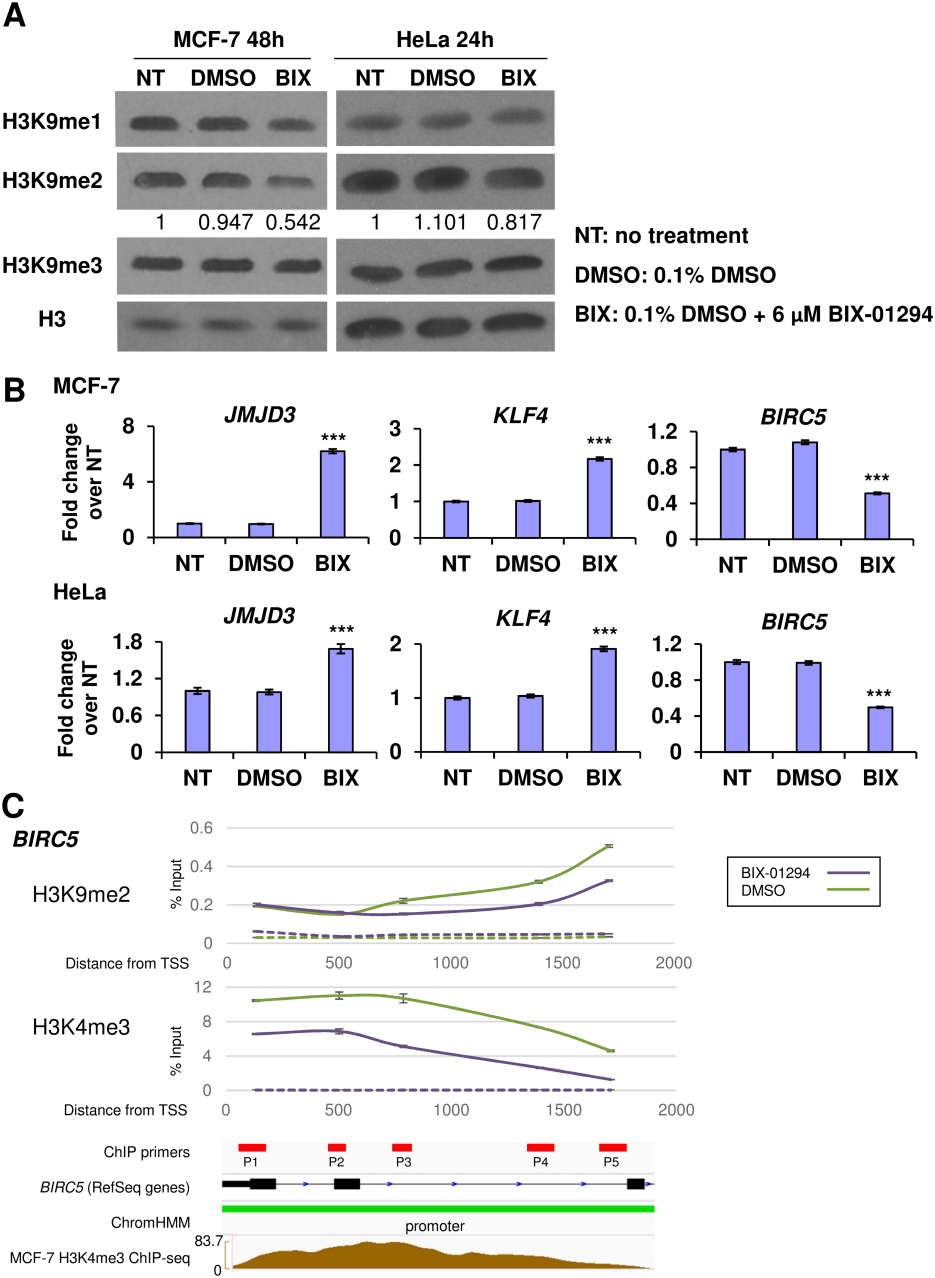
BIX-01294 attenuates H3K9 methylation and derepresses transcriptional regulation of target genes. (A) Western blots showing decrease in H3K9me1 (MCF-7) and H3K9me2 levels (MCF-7 and HeLa) after 48 and 24 hours respectively in the indicated treatments. Total histone 3 (H3) was used as the loading control. Numbers indicate band quantification of the relative levels of H3K9me2 to that of the NT control after normalization to H3. (B) Fold change in gene expression of *JMJD3*, *KLF4* and *BIRC5* in MCF-7 and HeLa cells treated with 6 μM BIX-01294 (BIX) compared to the NT and DMSO controls. Gene expression levels were normalized against the housekeeping reference gene *EEF1G* and fold change was calculated against the average of the NT controls. Error bars show SEM for n = 6 replicates. (C) ChIP-qPCR results showing enrichment of H3K4me3 and H3K9me2 around the TSS of *BIRC5* in MCF-7 cells treated with 6 μM BIX-01294 (purple) compared to the DMSO control (green). ChIP samples were normalized against the input control. Dotted lines show IgG controls for the respective treatments. Integrative Genomics Viewer (IGV) traces show the location of primers (red rectangles), exons (black rectangles), introns (connecting black lines with blue arrows indicating transcription direction), promoters (green rectangle) and H3K4me3 enrichment (brown) respectively for *BIRC5*.

As G9A functions as an H3K9 histone methyltransferase, any perturbation in G9A activity is expected to impact specifically upon its targeted epigenetic marks. To test the specificity of BIX-01294, we examined the changes in global H3K9 methylation in human breast and cervical cancer cells. The MCF-7 breast cancer cell line and the HeLa cervical cancer cell line were used as they both showed G9A upregulation compared to normal tissues (S1 Fig) and are consistent with the patient data from GENT (Fig 1A). We identified that the IC50 of BIX-01294 in MCF-7 cells was 4.222 μM after 24h under normoxic conditions and was slightly more potent at 3.349 μM in hypoxia (S2 Fig). At a concentration of 6 μM BIX-01294, similar to that administered in previous studies [16], global H3K9me2 was found to be significantly decreased in MCF-7 cells after 48h and in HeLa after 24h compared to untreated controls (Fig 2A), suggesting that the methyltransferase activity of G9A was blocked by BIX-01294. Notably, the loss of H3K9 methylation was limited predominantly to H3K9me2 with a small decrease in H3K9me1 in MCF-7 cells, in agreement with G9A function as it is also known to methylate H3K9me1 [8].

The change in H3K9 methylation status following G9A inhibition was expected to perturb the expression of G9A targets. Following BIX-01294 treatment, the expression of *JMJD3* and *KLF4*, shown to be upregulated in *G9a*-deficient cells in our previous study [6], and *BIRC5*, which we identified to be downregulated in the same study, was found to be similarly dysregulated in MCF-7 and HeLa cells (Fig 2B).

Finally, to determine if G9A inhibition leads specifically to changes in the H3K9 methylation status of the target genes themselves, we performed H3K9me2 ChIP-qPCR on *BIRC5*. *BIRC5* is an anti-apoptotic gene [28] and its downregulation may increase apoptosis in cancer cells, in line with the therapeutic effect of G9A inhibition. As expected with G9A inhibition, H3K9me2 enrichment was decreased on *BIRC5* compared to the control in MCF-7 cells (Fig 2C). To understand why *BIRC5* expression was downregulated with BIX-01294 although there was loss of the H3K9me2 silencing mark, we further performed ChIP-qPCR against H3K4me3 at regions of the *BIRC5* promoter known to have H3K4me3 enrichment [21]. Interestingly, H3K4me3 was also decreased (Fig 2C, S3A Fig), accounting for the reduced transcription of *BIRC5* despite the loss of H3K9me2. The expression of *BIRC5* is known to be regulated by transcriptional corepressors such as FOXO3/FKHRL1 and p53 [29,30], and their effects are determined by epigenetic modifications at the *BIRC5* promoter [31,32]. Therefore, these and possibly other corepressors may be responsible for the transcriptional downregulation of *BIRC5*. Nevertheless, we showed that BIX-01294 was indeed able to downregulate H3K9me2 in a gene- and site-specific manner reflective of the inhibition of G9A methyltransferase activity.

### G9A inhibition decreases cell migration and reduces anchorage-independent growth

**Fig 3:**
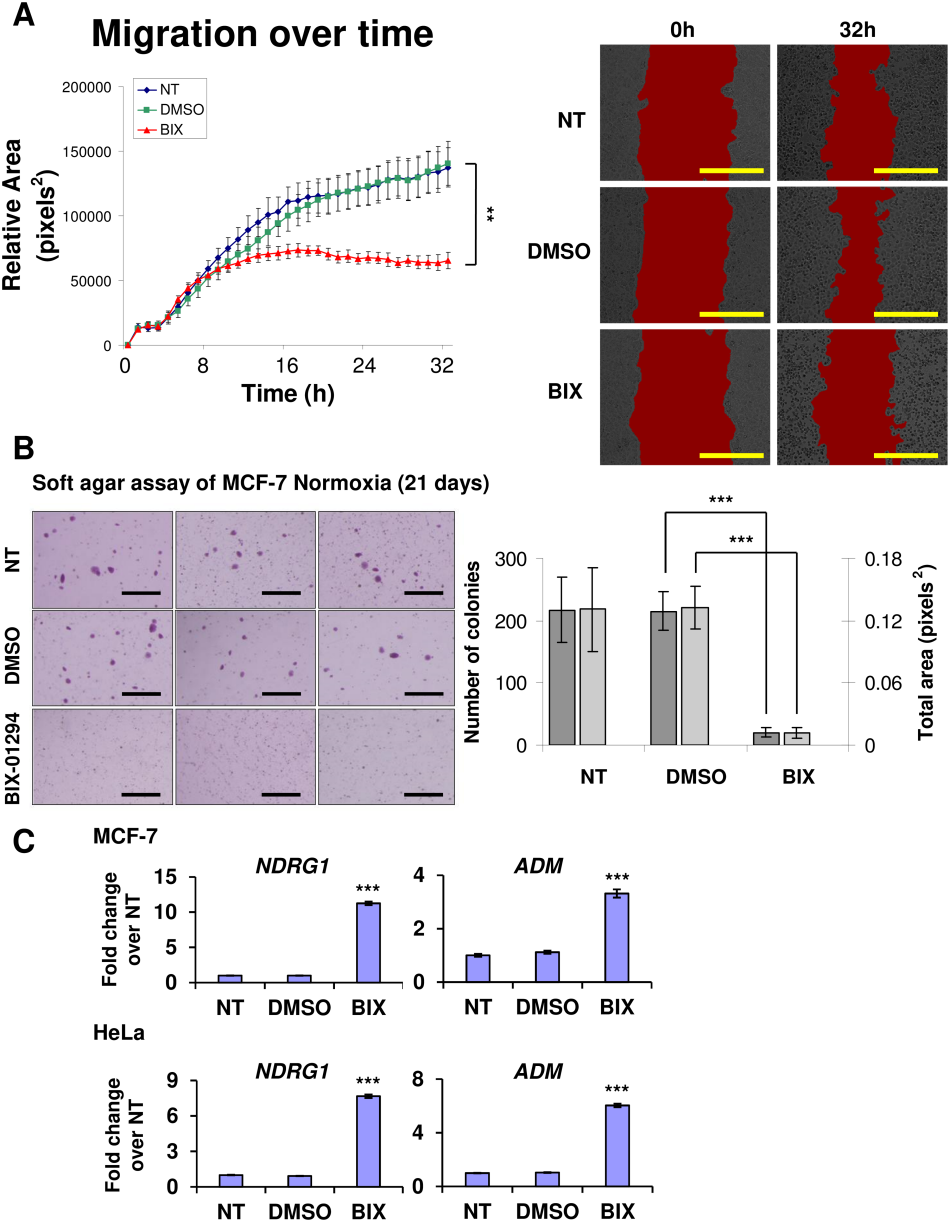
G9A inhibition leads to blockade of cell migration and loss of colony forming potential. (A) Graph showing migration area covered by MCF-7 cells treated with 6 μM BIX-01294 (BIX, red line) at hourly intervals over 32 hours in scratch wound healing assays compared to the controls (NT, blue line; DMSO, green line). Error bars indicate SEM for n = 3 replicates. Areas measured are demarcated in red in the representative micrographs at the start (0h) and end (32h) of the experiment respectively for each condition. Scale bars represent 500 μm. (B) Micrographs showing MCF-7 colony formation under the indicated conditions in soft agar assays. Micrographs shown are the most representative of the averages of n = 3 replicates. Scale bars denote 1 mm. Bar chart indicates the number of colonies (dark grey) and the total area of the colonies (light grey) formed from the MCF-7 cells in each respective treatment. Error bars indicate SEM for n = 3 replicates. (C) Fold change in gene expression of *NDRG1* and *ADM* in MCF-7 and HeLa cells treated with 6 μM BIX-01294 (BIX) compared to the NT and DMSO controls. Gene expression levels were normalized against the housekeeping reference gene *EEF1G* and fold change was calculated against the average of the NT controls. Error bars indicate SEM for n = 6 replicates.

Having shown that BIX-01294 was effective in curtailing G9A H3K9 methylation, we next tested the effects of the inhibitor in attenuating the oncogenic functions of G9A. G9A has been reported to drive metastasis by increasing cell migration and improving cell adhesion [33,34]. In scratch wound healing assays, BIX-01294 treatment decreased migration in MCF-7 cells (Fig 3A). Although migration rates were similar across all treatments over the initial 8 hours, the subsequent migration rate of cells treated with BIX-01294 slowed markedly, and showed complete arrest after 16 hours. Therefore, the time required for the effect of BIX-01294 to manifest on the migration of MCF-7 cells may occur in as little 8 hours, with full effects becoming visible after 16 hours, indicating the speed and efficacy of inhibiting G9A activity on migration function.

We next assessed the effect of G9A inhibition on cell adhesion using soft agar assays. BIX-01294 treatment significantly decreased the ability of MCF-7 to form colonies in soft agar (Fig 3B), suggesting the loss of anchorage-independent growth and colony forming potential with G9A inhibition.

The effects of BIX-01294 on cell migration and anchorage-independent growth may be due to the dysregulation of functionally associated genes. *KLF4*, which is known to inhibit colony formation and cell migration when overexpressed [35], was upregulated with BIX-01294 (Fig 2B). *NDRG1*, a tumor suppressor and metastasis blocker [36], and *ADM*, which has been reported to have anti-migratory effects [37], were also significantly upregulated with BIX-01294 (Fig 3C). *BIRC5*, which showed decreased expression with BIX-01294 (Fig 2B), can also inhibit cell migration when downregulated [38]. To independently validate the results with pharmacological inhibition of G9A, we performed *G9A* knockdowns in MCF-7 cells, which decreased growth rates of the cells, in agreement with *G9A* loss-of-function and the reduction of oncogenicity (S4A–S4C Fig). The knockdown effects on cell growth were not as strong as with BIX-01294 inhibition, possibly due to incomplete loss of G9A function with 20–30% residual *G9A* expression in the knockdowns. Together, these results demonstrate the therapeutic potential and effectiveness of G9A inhibition via BIX-01294 with the dysregulation of genes involved in cell migration and anchorage-independent growth.

### Cancer cells activate the HIF pathway in response to BIX-01294 inhibition of G9A

Interestingly, *JMJD3*, *KLF4*, *BIRC5*, *NDRG1* and *ADM*, which are dysregulated by BIX-01294, are also known to be hypoxia-regulated genes [24,39–41]. That, coupled with previous findings that G9a expression is regulated by hypoxia [4,6], suggests significant crosstalk between the hypoxia pathway and G9A in cancer cells.

**Fig 4:**
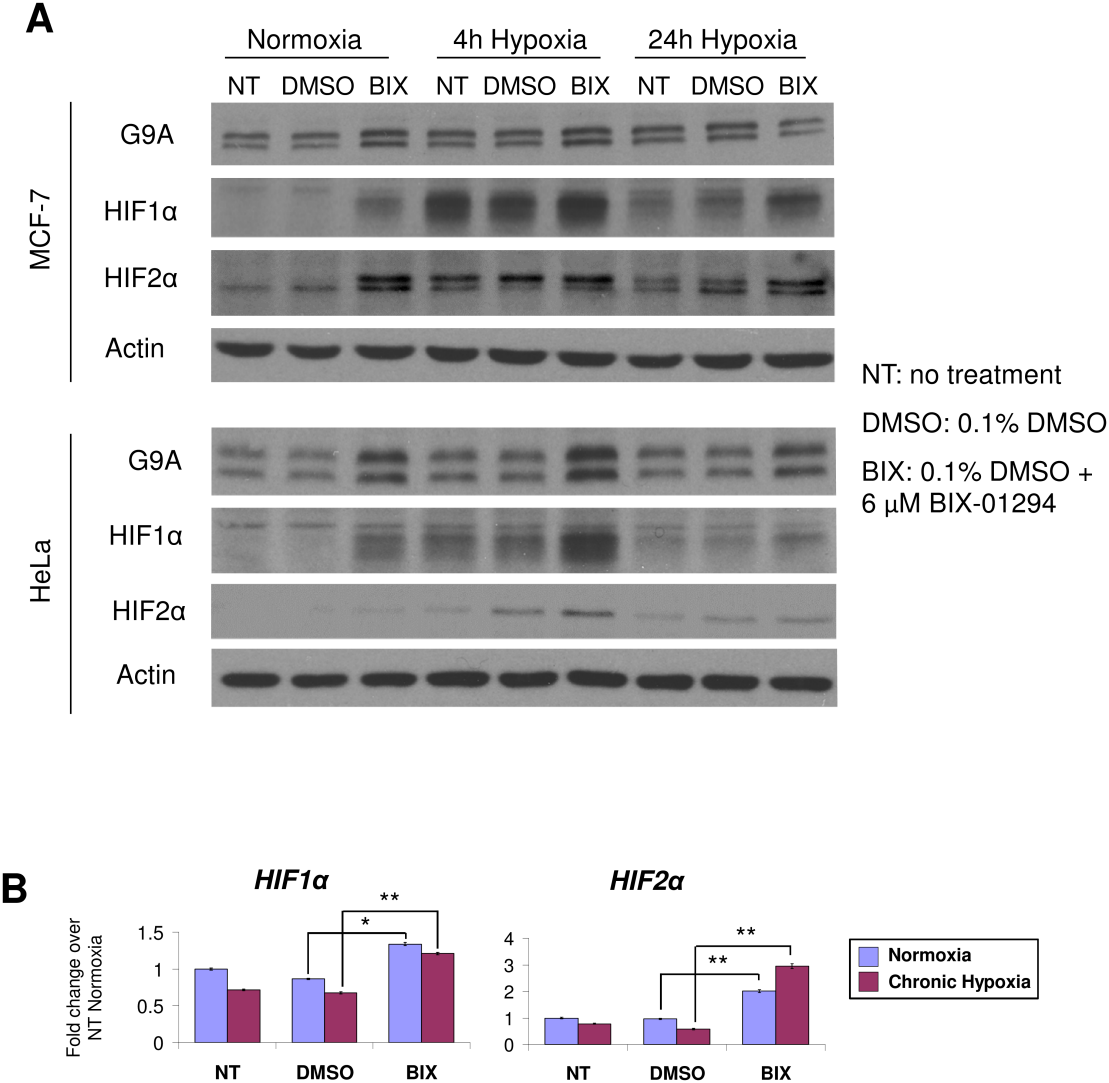
Cancer cells upregulate HIF1α and HIF2α in response to BIX-01294. (A) Protein levels of G9A, HIF1α and HIF2α in MCF-7 and HeLa cells after 24 hours under the indicated treatments. Actin was used as the loading control. (B) Fold change in gene expression of *HIF1α* and *HIF2α* in MCF-7 cells treated with 6 μM BIX-01294 (BIX) compared to the NT and DMSO controls in normoxia (blue) and 24 hours chronic hypoxia (magenta). Gene expression levels were normalized against the housekeeping reference gene *EEF1G* and fold change was calculated against the average of the NT controls in normoxia. Error bars indicate SEM for n = 9 replicates.

G9A protein levels were upregulated with hypoxia in both MCF-7 and HeLa cells (Fig 4A). Unexpectedly, the protein levels of HIF1α and HIF2α, the main effectors of the hypoxia pathway, were increased by BIX-01294 even in normoxia. Consistent with this, combined treatments of BIX-01294 with hypoxia led to a further increase of HIF protein levels that exceeded that of hypoxia alone, suggesting cancer cells can upregulate the HIFs independently over and above the effects of hypoxia in response to BIX-01294. Gene expression analyses of *HIF1α* and *HIF2α* in MCF-7 cells revealed a significant increase in transcript levels with BIX-01294 treatment in both normoxia and hypoxia (Fig 4B). Therefore, BIX-01294 may lead to the derepression of HIF transcription, causing the increase of HIF protein levels independently of hypoxia.

### G9A inhibition derepresses hypoxia target genes related to cell cycle, death and proliferation

**Fig 5:**
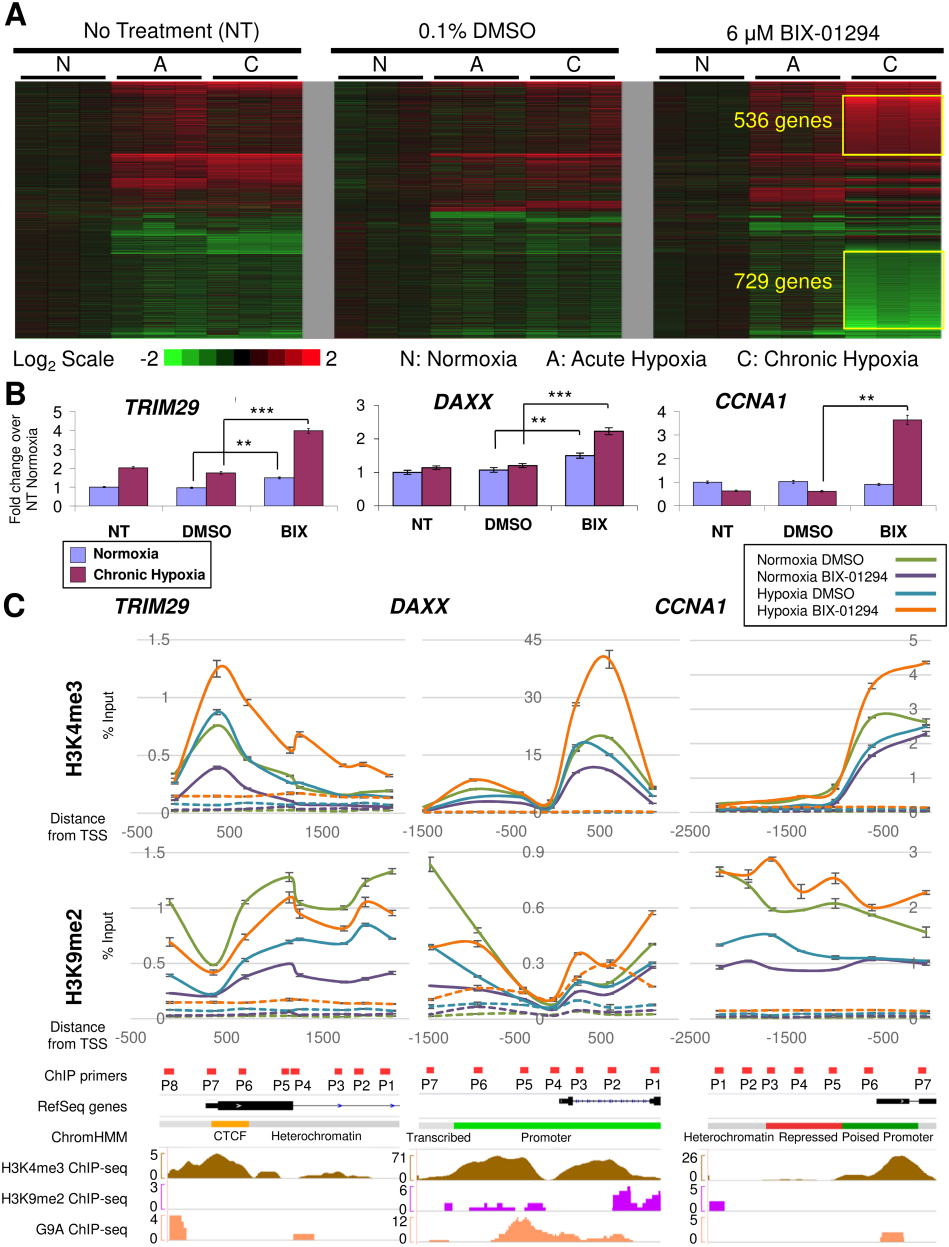
*De novo* derepression of hypoxia target genes with BIX-01294 and hypoxia treatments. (A) Global heatmap of gene expression changes in MCF-7 cells after 24 hours treatment with 6 μM BIX-01294 compared to the NT and DMSO controls in the indicated conditions (N, normoxia; A, 4 hours acute hypoxia; C, 24 hours chronic hypoxia). Gene expression was compared against the average of the normoxic control for each treatment and filtered for fold changes > 1.5 and *P* < 0.01. Color bar indicates fold change on a log_2_ scale in red for upregulation and green for downregulation. Genes that are *de novo* derepressed only with BIX-01294 and chronic hypoxia are indicated in yellow boxes. (B) Fold change in expression of *de novo* derepressed genes *TRIM29*, *DAXX* and *CCNA1* in MCF-7 cells treated with 6 μM BIX-01294 (BIX) compared to the controls (NT and DMSO) in normoxia (blue) and 24 hours chronic hypoxia (magenta). Gene expression levels were normalized against the housekeeping reference gene *EEF1G* and fold change was calculated against the average of the NT controls in normoxia. Error bars indicate SEM for n = 9 replicates. (C) ChIP-qPCR results showing enrichment of H3K4me3 and H3K9me2 around the TSS of *TRIM29*, *DAXX* and *CCNA1* in MCF-7 cells treated with 6 μM BIX-01294 in normoxia (purple) and 24 hours chronic hypoxia (orange) compared to the DMSO controls in normoxia (green) and 24 hours chronic hypoxia (blue). Enrichments were normalized against the input controls. Dotted lines show IgG controls for the respective treatments. IGV traces indicate location of primers (red rectangles), exons (black rectangles), introns (connecting black lines with blue arrows indicating transcription direction), CTCF, promoter and repressed regions (yellow, green and red rectangles respectively) for each gene.

We next carried out microarray studies to identify at the whole transcriptome level the hypoxia and G9A target genes that could account for the phenotypic effects on cancer function and may be candidates for cancer treatment, especially in the context of hypoxic tumors. A large number of genes became derepressed in chronic hypoxia after treatment with BIX-01294, with 536 and 729 genes becoming up- and downregulated respectively (Fig 5A). These *de novo* derepressed genes were termed as such since they were silenced and only responded to hypoxia when G9A was inhibited with BIX-01294, possibly due to the alleviation of the H3K9me2 repressive mark permitting formerly silenced hypoxia target genes to become regulated in their expression.

To determine which G9A targets in our microarray analysis were conserved with other G9A target gene datasets, we carried out a comparison against the microarray studies GSE22810 [10] and GSE41226 [33] deposited in NCBI GEO. GSE22810 contained expression data from both knockdown and overexpression of G9A in the human lung adenocarcinoma cell lines CL1-0 and CL1-5 while GSE41226 provided expression data from two G9A knockdowns in the ovarian cancer cell line SKOV3. We found that, out of the 536 *de novo* derepressed genes that were upregulated by hypoxia following G9A inhibition in our study, 80 genes (14.9%) were also upregulated in these other G9A microarray datasets (1 in both GSE22810 and GSE41226, 20 in GSE22810 only and 59 in GSE41226 only). The remaining 456 genes (85.1%) are G9A targets upregulated specifically in our microarray dataset (S5A Fig). Likewise, out of the 729 *de novo* derepressed genes identified to be downregulated by hypoxia following G9A inhibition in our study, 41 genes (5.62%) were also downregulated in the other G9A microarray studies (2 in both GSE22810 and GSE41226, 2 in GSE22810 only and 37 in GSE41226 only), while the remaining 688 genes (94.4%) are G9A targets downregulated only in our dataset. The majority of the *de novo* derepressed genes from our study have not been previously identified as G9A targets and are dysregulated only with both hypoxia and G9A inhibition, indicating that the derepression of hypoxia targets by G9A inhibition is indeed a novel phenomenon.

In addition to looking at the genes that are *de novo* derepressed and rendered hypoxia-responsive by G9A inhibition, we also examined the genes that were dysregulated by BIX-01294 alone. 219 out of 1280 (17.1%) of the upregulated and 74 out of 1390 (5.32%) of the downregulated targets were found to be overlapping with the G9A targets described in the datasets GSE22810 and GSE41226 (S5B Fig). Consequently, some of the target genes we identified in our microarray study are indeed consistent with the G9A targets described in other studies, thereby conferring greater confidence in the reliability of our dataset.

Through gene ontology analyses, we found that cell death and survival, cell cycle and cellular growth and proliferation were among the top functional categories that the *de novo* upregulated genes were associated with (S5C Fig). The expression of these genes, including *TRIM29*, *DAXX* and *CCNA1*, was consistently derepressed and dysregulated in chronic hypoxia and BIX-01294 treatment when validated by RT-qPCR (Fig 5B, S5D Fig). *TRIM29* decreases apoptosis and promotes cell growth and metastasis [42,43]. *DAXX* has anti-apoptotic functions and can cause transcriptional repression [44]. *CCNA1* has been reported to enable cells to bypass G1 arrest when overexpressed, conferring a growth advantage to these cells [45]. Some of these genes were found to be consistently derepressed when *G9A* was knocked down and acquired an enhanced response to hypoxia, further validating the specificity of BIX-01294 in inhibiting G9A with the loss of H3K9me2-mediated silencing (S5E Fig). Together, the gene ontology results with the validation of survival and cell cycle genes suggest that HIF upregulation and the newly acquired sensitivity of target genes to the HIFs when derepressed by G9A inhibition may trigger a survival response in the breast cancer cells examined.

To evaluate if these genes were dysregulated due to the inhibition of G9A’s methyltransferase activity by BIX-01294, the levels of the H3K9me2 repressive and H3K4me3 activating marks around the transcription start sites (TSS) of these genes were analysed by ChIP-qPCR. Using MCF-7 ChIP-sequencing (ChIP-seq) datasets from ENCODE and NCBI GEO [21,22], regions with high enrichments for H3K4me3 were identified (S3B–S3D Fig). However, no significant enrichments (signal > 5 read counts) could be detected for H3K9me2 for the genes examined in the datasets.

In our ChIPs, both H3K9me2 and H3K4me3 enrichment at these regions could be detected specifically compared to the IgG controls in hypoxia and during G9A inhibition (Fig 5C). The H3K4me3 enrichment profiles and distributions were found to be experimentally matching with the ChIP-seq datasets, confirming that the ChIP results were specific and reproducible. In normoxia, BIX-01294 decreased H3K4me3 levels in all 3 genes compared to the DMSO-treated controls. For *TRIM29* and *DAXX*, the decrease in H3K4me3 was inconsistent with the rise in gene expression (Fig 5B), suggesting that more complex transcriptional regulation by other epigenetic or transcription factors may be at work in normoxia. However, combined treatments of hypoxia and BIX-01294 increased H3K4me3 levels beyond that of the DMSO controls for all 3 genes which is indicative of gene activation, largely consistent with the gene expression changes in these *de novo* derepressed genes.

When we examined the repressive H3K9me2 mark in all 3 genes, this was decreased when cells were treated with BIX-01294 alone. Notably, combined treatment with BIX-01294 and hypoxia restored H3K9me2 levels near to or beyond those of the normoxia DMSO controls, suggesting that hypoxia may have opposing effects against BIX-01294. Furthermore, significant enrichment for G9A could be identified at the promoter of some target genes, especially *DAXX* (Fig 5C, S3A–S3D Fig) [23], although enrichment levels were low in these ChIP datasets. We also examined HIF ChIP-seq datasets but these did not show high efficiency pulldowns for these regions (S3A–S3D Fig) [24].

In conclusion, G9A inhibition by BIX-01294, while effective in reducing H3K9me2 at target genes, also reflected the unexpected consequences of hypoxia pathway activation. Hypoxia was able to increase H3K9me2 against BIX-01294 possibly through the regulation of G9A and other histone methyltransferases or demethylases, although the mechanisms remain unclear. As the genes examined are involved in cell cycle and survival, this may be indicative of a survival response mounted by cancer cells against BIX-01294’s curtailment of cellular growth and oncogenic function.

### BIX-01294 continues to drive apoptosis and reduce clonogenicity in hypoxia, but hypoxia may partially rescue cell cycle arrest

**Fig 6:**
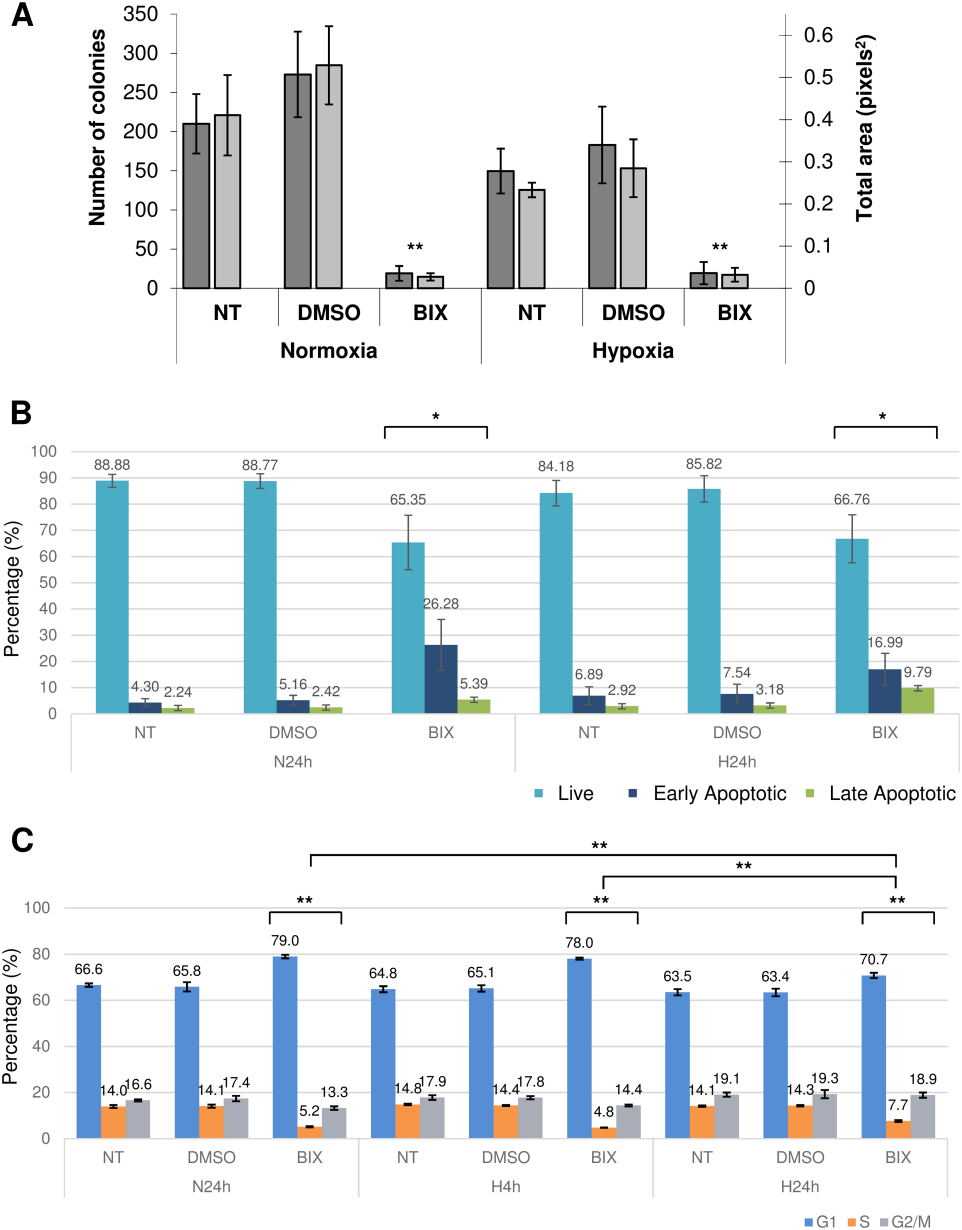
Hypoxia improves survival against BIX-01294’s inhibition of G9A by antagonizing cell cycle arrest. (A) Bar chart indicating the number of colonies (dark grey) and the total area of the colonies (light grey) in soft agar colony forming assays of MCF-7 cells in the indicated conditions. Error bars indicate SEM for n = 3 replicates. (B) Apoptosis analysis showing the distribution of live, early apoptotic and late apoptotic MCF-7 cells when treated with 6 μM BIX-01294 (BIX) compared to the controls (NT and DMSO) in 24 hours normoxia (N24h) and 24 hours chronic hypoxia (H24h). Bar chart indicates the percentage of live (light blue), early apoptotic (dark blue) and late apoptotic (green) cells out of the total number of cells for each treatment. Error bars indicate SEM for n ≥ 6 replicates. (C) Cell cycle analysis showing the distribution of MCF-7 cells in different cell cycle phases. Bar chart indicates the percentage of cells in the G1 (blue), S (orange) and G2/M (grey) phases of cell cycle out of the total number of cells for each treatment (NT, untreated; DMSO, 0.1% DMSO; BIX, 0.1% DMSO + 6 μM BIX-01294; N24h, 24 hours normoxia; H4h, 4 hours acute hypoxia; H24h, 24 hours chronic hypoxia). Error bars indicate SEM for n = 3 replicates.

We next performed functional assays to link the *de novo* derepressed response of the hypoxia pathway with the biological and phenotypic effects manifested in the breast cancer cells. Previously, our clonogenic assays showed that BIX-01294 treatment was able to inhibit colony formation on soft agar, indicative of decreased anchorage-independent growth (Fig 3B). In hypoxia, BIX-01294 continued to reduce the colony forming potential of cells, significantly reducing both the number and total area of the colonies formed compared to the controls (Fig 6A). There was no significant difference in hypoxia alone compared to normoxia, suggesting that hypoxia does not play a significant role in improving the clonogenicity of the breast cancer cells against BIX-01294’s eradication of the colonies.

Next, we carried out assays to assess the level of apoptosis using Annexin V and SYTOX Blue viability dye staining. BIX-01294 induced significant apoptosis in MCF-7 cells under both normoxia and hypoxia. The proportions of both late (stained for Annexin V and SYTOX Blue) and early (stained for Annexin V only) apoptotic cells were significantly increased in the BIX-01294-treated cells compared to the controls (Fig 6B, S6A Fig). The proportion of live cells, which do not stain with either dye, showed a corresponding decrease with BIX-01294 compared to the controls. In hypoxia, there was a reduction in the proportion of the early apoptotic population after treatment with BIX-01294 and a corresponding increase in the late apoptotic population compared to that of normoxia, but this difference was not statistically significant.

Finally, in our cell cycle analysis, we found a significant increase in the proportion of cells in the G1 phase and a corresponding decrease in those of the S and G2/M phases with BIX-01294 treatment compared to the controls (Fig 6C, S6B Fig). This indicates that G9A inhibition mediates cell cycle arrest in the G1 phase, preventing cells from entering the S and G2/M phases. The G1 phase cell cycle arrest with BIX-01294 treatment also occurred under hypoxic conditions. However, chronic hypoxia was able to partially rescue the proportion of cells in G1 phase arrest and increase those in the S and G2/M phases compared to normoxia and acute hypoxia. This statistically significant effect was antagonistic to the G1 phase cell cycle arrest induced by BIX-01294 treatment alone.

In conclusion, despite the activation of the hypoxia pathway in breast cancer cells, BIX-01294 was still able to induce apoptosis and cell cycle arrest. However, the alleviation of G1 cell cycle arrest induced by BIX-01294 in chronic hypoxia was statistically significant, suggesting that chronic hypoxia may attenuate the therapeutic effect of BIX-01294 through cell cycle regulation. Although G9A inhibition was effective in curtailing oncogenic functions, it may be beneficial to inhibit the HIF pathway in combinatorial therapies to mitigate the survival of some cancer cells that may subsequently lead to relapse and the development of chemoresistance.

### HIF inhibition antagonizes the response of *de novo* hypoxia targets derepressed by BIX-01294

**Fig 7:**
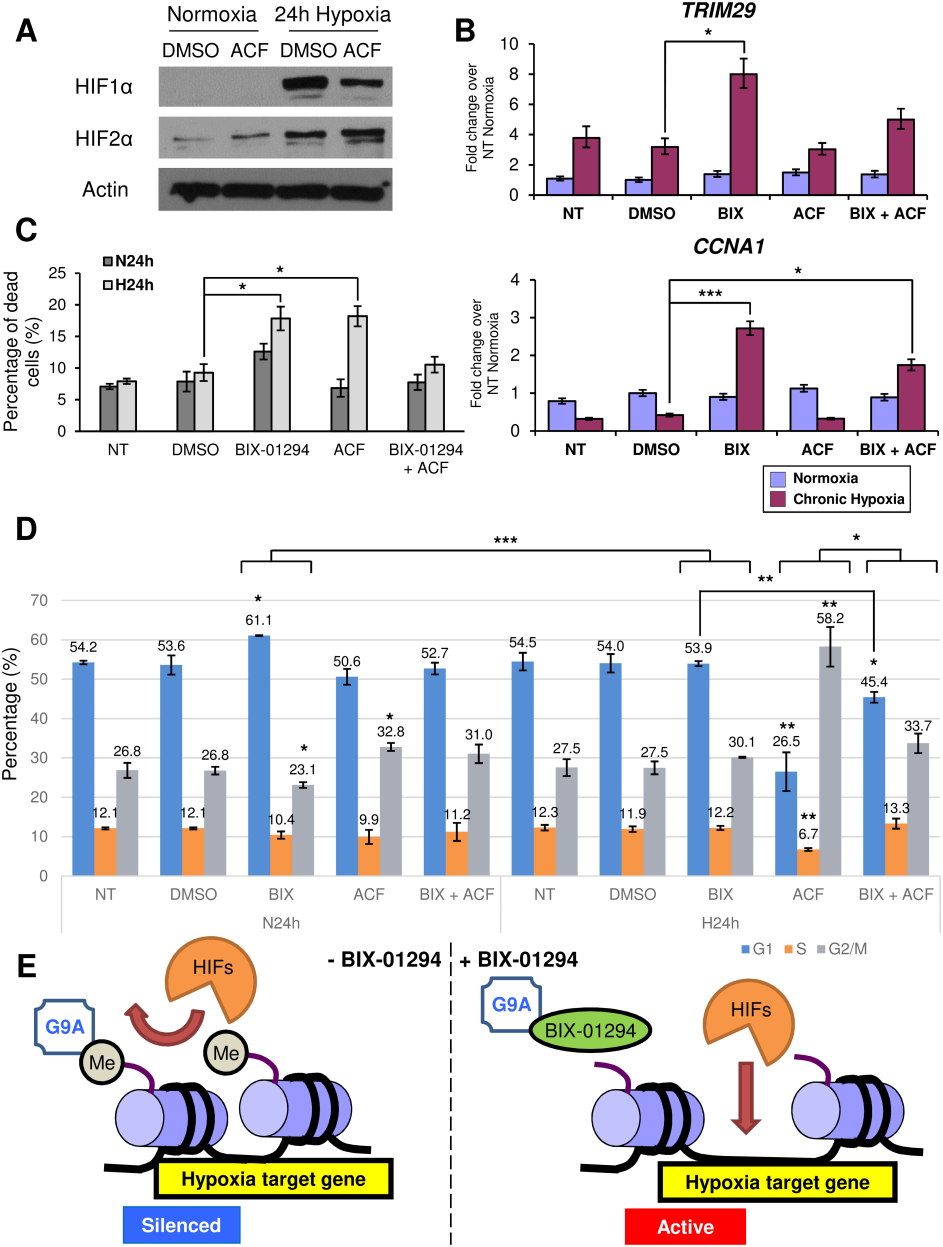
HIF inhibition by ACF antagonizes the response of *de novo* hypoxia targets derepressed by G9A inhibition to chronic hypoxia. (A) Western blots show protein levels of HIF1α and HIF2α in MCF-7 cells after 24 hours under the indicated treatments. Actin was used as the loading control. (B) Fold change in gene expression of *TRIM29* and *CCNA1* in MCF-7 cells treated with 6 μM BIX-01294 (BIX) and/or 0.6 μM ACF compared to the NT and DMSO controls in normoxia (blue) and 24 hours chronic hypoxia (magenta). Gene expression levels were normalized against the housekeeping reference gene *EEF1G* and fold change was calculated against the average of the NT controls in normoxia. Error bars indicate SEM for n = 9 replicates. (C) Bar chart shows percentage of dead cells staining positive for trypan blue following treatment of MCF-7 cells with the compounds as indicated under 24 hours normoxia (N24h) and 24 hours chronic hypoxia (H24h). Error bars denote SEM for n = 3 replicates. (D) Cell cycle analysis showing the distribution of MCF-7 cells in different cell cycle phases. Bar chart indicates the percentage of cells in the G1 (blue), S (orange) and G2/M (grey) phases of cell cycle out of the total number of cells for each treatment (NT, untreated; DMSO, 0.1% DMSO; BIX, 0.1% DMSO + 6 μM BIX-01294; ACF, 0.1% DMSO + 0.6 μM ACF; N24h, 24 hours normoxia; H24h, 24 hours chronic hypoxia). Error bars indicate SEM for n = 3 replicates. (E) Schema showing the effect of G9A inhibition by BIX-01294 on hypoxia target gene activation. In the absence of BIX-01294 (left), G9A methylates (Me) H3K9, causing the chromatin to maintain a closed conformation characteristic of silenced genes and preventing the HIFs from transcriptionally regulating their target genes. With BIX-01294 (right), G9A is inhibited and unable to methylate H3K9, allowing the chromatin structure to open and the HIFs to regulate these derepressed hypoxia target genes.

To test the hypothesis that the activation of HIF signaling could compromise the anti-cancer effect of BIX-01294 in chronic hypoxia, we used acriflavine (ACF), which is known to inhibit HIF1α and 2α [46] and has shown therapeutic potential for use against chronic myeloid leukemia [47]. When MCF-7 cells were treated with ACF at its hypoxic IC50 of 0.6 μM (S7A Fig) in chronic hypoxia, HIF1α protein levels were successfully decreased (Fig 7A).

We next investigated the effect of ACF on the expression of the *de novo* derepressed genes responsive to chronic hypoxia and BIX-01294 treatment. Interestingly, the inhibition of the HIF pathway by ACF attenuates the hypoxic induction of these genes (Fig 7B, S7B Fig), suggesting these *de novo* derepressed genes are indeed HIF targets.

In trypan blue exclusion assays, although BIX-01294 and ACF were able to reduce cell viability and increase cell death in hypoxia individually, the combination of BIX-01294 and ACF was able to rescue cell viability and reduce cell death to levels comparable to that of the controls (Fig 7C), indicating that the HIFs may be required for the therapeutic effect of BIX-01294.

Furthermore, cell cycle analysis on MCF-7 cells treated with ACF showed that ACF was able to significantly increase the G2/M population in normoxia (Fig 7D). In hypoxia, the G2/M population was dramatically increased, with a corresponding decrease in both the G1 and S phases. The decrease in the G1 population coupled with G2 arrest following ACF treatment suggests that the HIFs may not only contribute to G1 phase arrest but may also be important for cell cycle progression.

The combined treatment of both ACF and BIX-01294 in hypoxia resulted in a moderation of the effect of both inhibitors, with a significant decrease of the G1 population compared to cells treated with only BIX-01294 and a corresponding significant increase compared to cells treated with only ACF. The combined treatment also showed that BIX-01294 could reverse the effects of ACF, fully rescuing the decrease in the S phase population and increase in the G2/M phase population. The results of the combined treatment demonstrate that HIF inhibition moderates the effect of BIX-01294, further illustrating that the HIFs may facilitate the effects of G9A inhibition.

We thus propose the following model by which G9A and hypoxia are able to interact in gene regulation (Fig 7E). G9A, which is overexpressed in cancers, aberrantly methylates H3K9 at the promoters of numerous genes. This causes the chromatin to adopt a closed heterochromatin conformation, leading to the repression of genes and preventing transcription factors such as the HIFs from reaching their targets, therefore blocking the regulation of gene expression. However, when BIX-01294 is used in cancer cells, loss of G9A function leads to reduced H3K9 methylation on the promoters of these genes, allowing the chromatin to become accessible and the previously silenced genes to become responsive to transcriptional regulation. As demonstrated in our study, many of these *de novo* derepressed genes are involved in functions such as cell death and survival, cell cycle and cellular growth and proliferation, and may contribute to decreased colony formation, apoptosis, and the dysregulation of cell cycle in a HIF-dependent manner. G9A inhibition may thus prove to be a potential therapy by which aberrantly silenced genes may be reactivated for therapeutic outcomes in breast cancer cells.

## Discussion

There has been growing interest in the exploitation of epigenetic mechanisms as viable targets of cancer therapy [48,49]. G9A is a key therapeutic target as it can regulate downstream genes via epigenetic mechanisms to promote cancer, such as the silencing of the tumor suppressor *RUNX3* in gastric cancer [5]. We have shown negative effects on prognosis (Fig 1B–1D) where high *G9A* expression portends poorer survival in breast cancer patients, particularly those with ER+ subtypes. Our finding that *G9A* overexpression occurs across a broad spectrum of cancers (Fig 1A) suggests that G9A inhibition as a form of cancer therapy may be applicable to other cancers besides breast malignancies. Indeed, the independent HeLa cervical cancer cell line also showed reduction of H3K9me2 methylation with BIX-01294 (Fig 2A), dysregulation of the same target genes as MCF-7 (Figs 2B and 3C) and activation of the HIF pathway independently of hypoxia (Fig 4A).

Although BIX-01294 inhibition of G9A in normoxia reduced H3K9me2 both globally (Fig 2A) and at the gene specific level (Figs 2C and 5C), consistent with G9A loss-of-function, we found that loss of the repressive H3K9me2 mark does not directly translate to upregulated gene expression. *BIRC5* was downregulated (Fig 2B) and in our microarray profiling, more than half (57.6% or 729 genes) of the *de novo* derepressed target were also downregulated (Fig 5A). This may be due to the function of H3K9me2 as a silencing mark, where it induces gene repression via heterochromatin condensation [12–14]. Upon derepression with the loss of H3K9me2 during BIX-01294 treatment, the assembly of either transcriptional activators or repressors drives the up- or downregulation of gene expression respectively. Therefore, the H3K9me2 mark is not the sole determinant of gene expression, which may also depend on whether transcriptional activators or repressors are recruited. Indeed, the transcriptional activation mark H3K4me3 regulates the change in gene expression alongside H3K9me2 and is therefore an additional determinant of transcriptional activity.

BIX-01294 was able to reduce migration (Fig 3A), inhibit anchorage-independent growth (Figs 3B and 6A), increase apoptosis (Fig 6B) and cause cell cycle arrest (Fig 6C), consistent with other independent studies where *G9A* knockdown also inhibited cell migration and metastasis in breast cancer cells [34] and induced G1 phase cell cycle arrest [50,51]. This suggests that the effects of BIX-01294 on oncogenic processes are specific and reproducible with other G9A loss-of-function experiments and, together with our previous findings that BIX-01294 can reduce tumor growth *in vivo* [6], make BIX-01294 and other G9A inhibitors of strong clinical interest as pharmacological agents.

Unexpectedly, G9A inhibition was able to activate the HIF pathway even in normoxia (Fig 4A and 4B) and derepress *de novo* hypoxia target genes (Fig 5A). This is in contrast to a previous study by Oh *et al*. which reported that HIF1α is destabilized by BIX-01294 in HepG2 hepatoma cells [52], suggesting that the regulation of the HIFs by G9A inhibition may occur in a context-dependent manner that varies with cell type. BIX-01294 was also administered at a significantly lower concentration of 1 μM, which induced no observable cell death, compared to our study, where 6 μM was used. This difference in BIX-01294 concentration may trigger a different set of signaling pathways and effects in the cells and may account for the variable effects on HIF1α protein levels. Nevertheless, both our and the Oh *et al*. studies have concluded that the inhibition of G9A by BIX-01294 leads to anti-tumorigenic effects and therefore has therapeutic potential.

While G9A inhibition remains effective in managing the growth of cancer cells, the accompanying activation of the HIF pathway raises the possibility that this may reduce the therapeutic effect of BIX-01294. As the HIF pathway is linked to improved survival, increased metastasis and heightened aggressiveness of cancer cells [2,3], its activation may be a mitigating response to cellular stress induced by drugs such as BIX-01294 as they drive cell cycle arrest and apoptosis. Cellular stress induced by radiotherapy and ROS accumulation has also been reported to activate the HIF pathway even in normoxia [53,54].

To investigate the role of the hypoxia pathway and its association with G9A inhibition, ACF, a known inhibitor of the HIFs [46], was used in conjunction with BIX-01294. ACF, contrary to the effects of BIX-01294, released cells from G1 arrest and instead, induced a G2 phase arrest. It has been reported that HIF1α activates cell cycle regulators such as p21 and p27 [55,56], hence it is not surprising that HIF inhibition releases cells from G1 arrest. We also demonstrated that ACF attenuates the hypoxic response of the *de novo* hypoxia targets derepressed by BIX-01294 at the gene expression level (Fig 7B, S7B Fig), suggesting that HIF activation may be essential in the regulation of these target genes.

We have therefore provided insights into the crosstalk and interaction between the epigenetic regulator G9A and the hypoxia pathway in cancer, as well as the potential therapeutic effect that BIX-01294 may have (Fig 7B–7E). Our findings highlight the utility of G9A inhibition in addressing the growth of cancer cells and have exciting implications on the use of BIX-01294 and other epigenetic inhibitors in cancer therapy, especially in the context of solid tumors which are highly heterogeneous and frequently hypoxic. Further studies to elucidate the interaction between the hypoxia pathway and the epigenome will be crucial for the development and refinement of epigenetic targeting strategies for cancer treatments.

## Supporting information

S1 Fig G9A expression across normal tissues, patient cancer tissues and cell lines in breast and cervix from the Genevestigator database. Box plots show the level of *G9A* expression on a log_2_ scale. *G9A* is upregulated in breast and cervical cancer patient tissue and cell lines compared to normal tissues. The number of samples examined in each cell and tissue type are indicated on the right.
(TIF)

S2 Fig IC50s of BIX-01294 under normoxic and hypoxic conditions. Graphs showing the normalized viability of MCF-7 cells after treatment with up to 50 μM BIX-01294 over 24 hours. The IC50 of BIX-01294 as determined by MTS was 4.222 μM in normoxia and 3.349 μM in hypoxia. Error bars indicate SEM for n = 6 replicates.
(TIF)

S3 Fig Identification of G9A, H3K4me3, H3K9me2, HIF1α and HIF2α binding sites in the loci of BIX-01294 responsive target genes. IGV profiles indicate location of primers (red rectangles), exons (black rectangles), introns (connecting black lines with blue arrows indicating direction of transcription), promoter, CTCF, enhancer and repressed regions (green, yellow, blue and red rectangles respectively), and enrichment for H3K4me3 (brown), H3K9me2 (magenta), G9A (orange) and HIF1α and HIF2α (light and dark blue respectively) for (A) *BIRC5*, (B) *TRIM29*, (C) *DAXX* and (D) *CCNA1*.
(TIF)

S4 Fig Knockdown of *G9A* reduces proliferation of MCF-7 breast cancer cells. (A) Western blots showing the decrease in G9A protein levels in MCF-7 cells expressing five independent *G9A* shRNAs (#1 to #5) compared to the control shRNA knockdown (Ctrl) and the untreated wild-type control (WT). Actin was used as the loading control. (B) Fold change of *G9A* expression in five independent *G9A* shRNA knockdowns (#1 to #5) compared to the Ctrl and WT controls. Gene expression levels were normalized against the housekeeping reference gene *EEF1G* and fold change was calculated against the average of the WT controls in normoxia. Error bars indicate SEM for n = 9 replicates. (C) Bar chart showing a significantly lower number of *G9A* shRNA #1 and #3 knockdown MCF-7 cells after 72 hours (Day 3, light grey) from an initial seeding of 2 x 10^5^ cells (Day 0, dark grey) compared to that of the Ctrl and WT (*P* < 0.05). Error bars indicate SEM for n = 3 replicates.
(TIF)

S5 Fig Derepression of target genes occurs in both G9A inhibition and *G9A* knockdown, enhancing their response to hypoxia. (A) Pie charts show the number of up- and downregulated *de novo* derepressed genes identified to also be dysregulated in the G9A microarray studies GSE22810 and GSE41226. (B) Pie charts show the number of BIX-01294 up- and downregulated genes identified to also be dysregulated in the G9A microarray studies GSE22810 and GSE41226. (C) IPA gene ontology analysis of up- and downregulated *de novo* derepressed genes in chronic hypoxia with BIX-01294 treatment that are differentially expressed by at least 1.5-fold over the average of the normoxic cells in BIX-01294. The top eight biological functions are shown, with a cut-off of *P* = 0.05 for Fisher's exact test (red lines). (D) Fold change in expression of *SH3GL3*, *SFRS7* and *SFXN2* in MCF-7 cells treated with 6 μM BIX-01294 (BIX) compared to the NT and DMSO controls in normoxia (blue) and 24 hours chronic hypoxia (magenta). Gene expression levels were normalized against the housekeeping reference gene *EEF1G* and fold change was calculated against the average of the NT controls in normoxia. Error bars indicate SEM for n = 9 replicates. (E) Fold change in expression of *TRIM29*, *SH3GL3*, *SFRS7* and *SFXN2* in MCF-7 cells expressing *G9A* shRNAs #1 and #3 compared to the control shRNA knockdown (Ctrl) and the untreated WT control (WT) in normoxia (blue) and 24 hours chronic hypoxia (red). Gene expression levels were normalized against the housekeeping reference gene *EEF1G* and fold change was calculated against the average of the WT controls in normoxia. Error bars indicate SEM for n = 9 replicates.
(TIF)

S6 Fig BIX-01294 continues to drive apoptosis in hypoxia, but hypoxia partially rescues cell cycle arrest induced by BIX-01294. (A) Apoptosis analysis with Annexin V and SYTOX Blue stains showing the distribution of live, early apoptotic and late apoptotic MCF-7 cells treated with 6 μM BIX-01294 (BIX) compared to the no treatment and DMSO controls in normoxia and 24 hours chronic hypoxia (Hypoxia 24h). The x-axis shows fluorescence intensity from Annexin V staining indicative of cells undergoing apoptosis, while the y-axis shows SYTOX blue fluorescence, indicative of dead cells. FACS images shown are the most representative of the averages of n ≥ 6 replicates. (B) Cell cycle analysis showing the distribution of MCF-7 cells in the G1 (P4), S (P5) and G2/M (P6) phases when treated with 6 μM BIX-01294 compared to the no treatment and DMSO controls in normoxia, 4 hours acute hypoxia (Hypoxia 4h) and 24 hours chronic hypoxia (Hypoxia 24h). The x-axis shows PI fluorescence intensity, while the y-axis shows the cell count. FACS images shown are the most representative of the averages of n = 3 replicates.
(TIF)

S7 Fig HIF inhibition by ACF attenuates the hypoxic regulation of *de novo* derepressed hypoxia targets. (A) Graph shows the normalized viability of MCF-7 cells after treatment with up to 100 μM ACF over 24 hours. The IC50 of ACF as determined by MTS was 2.126 μM in normoxia and 0.5655 μM in hypoxia. Error bars indicate SEM for n = 6 replicates. (B) Fold change in gene expression of *SH3GL3* and *SFRS7* in MCF-7 cells treated with 6 μM BIX-01294 (BIX) and/or 0.6 μM ACF compared to the NT and DMSO controls in normoxia (blue) and 24 hours chronic hypoxia (magenta). Gene expression levels were normalized against the housekeeping reference gene *EEF1G* and fold change was calculated against the average of the NT controls in normoxia. Error bars indicate SEM for n = 9 replicates. (C) Histograms show cell cycle analysis with the distribution of MCF7 cells in the G1, S and G2/M phases when treated with 6 μM BIX-01294 and/or 0.6 μM ACF compared to the no treatment and DMSO controls in normoxia and 24 hours chronic hypoxia (Hypoxia 24h). The percentages of cells out of the total that are in G1, S and G2/M phases are indicated. The x-axis shows PI fluorescence intensity, while the y-axis shows the cell count. FACS images shown are the most representative of the averages of n = 3 replicates.
(TIF)

S1 Table Primers used for RT-qPCR analysis of gene expression. (XLSX)

S2 Table Sequences used for *G9A* knockdown. (XLSX)

S3 Table Primers used for ChIP-qPCR analysis. (XLSX)
