# Supplementary figures and images for: Inhibition of the H3K9 methyltransferase G9A attenuates oncogenicity and activates the hypoxia signaling pathway
Source: PLoS One. 2017 Nov 16;12(11):e0188051. doi: 10.1371/journal.pone.0188051 (PMC5690420; doi:10.1371/journal.pone.0188051)

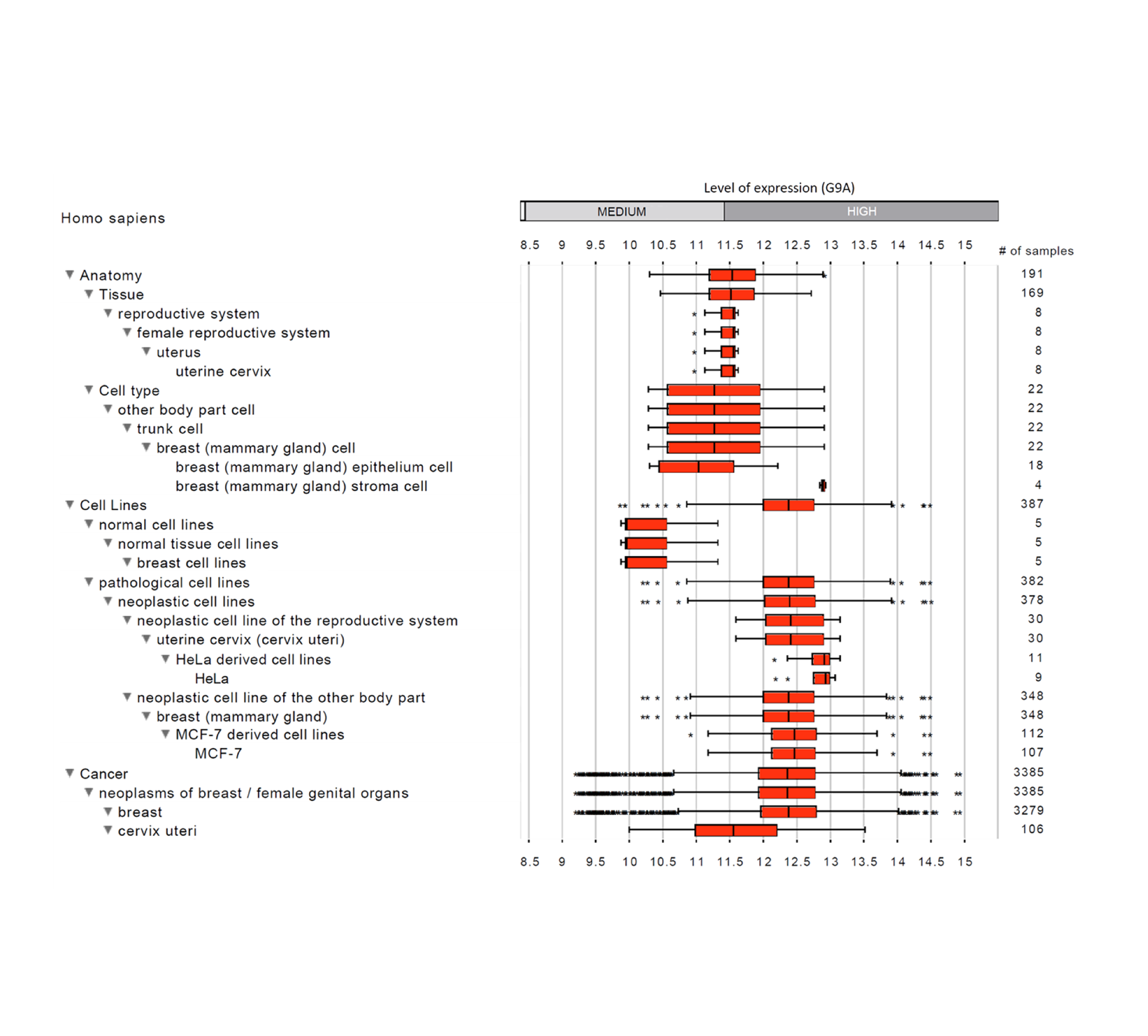

Supplement: S1 Fig — Box plots show the level of G9A expression on a log2 scale. G9A is upregulated in breast and cervical cancer patient tissue and cell lines compared to normal tissues. The number of samples examined in each cell and tissue type are indicated on the right. (TIF) [file pone.0188051.s001.tif]

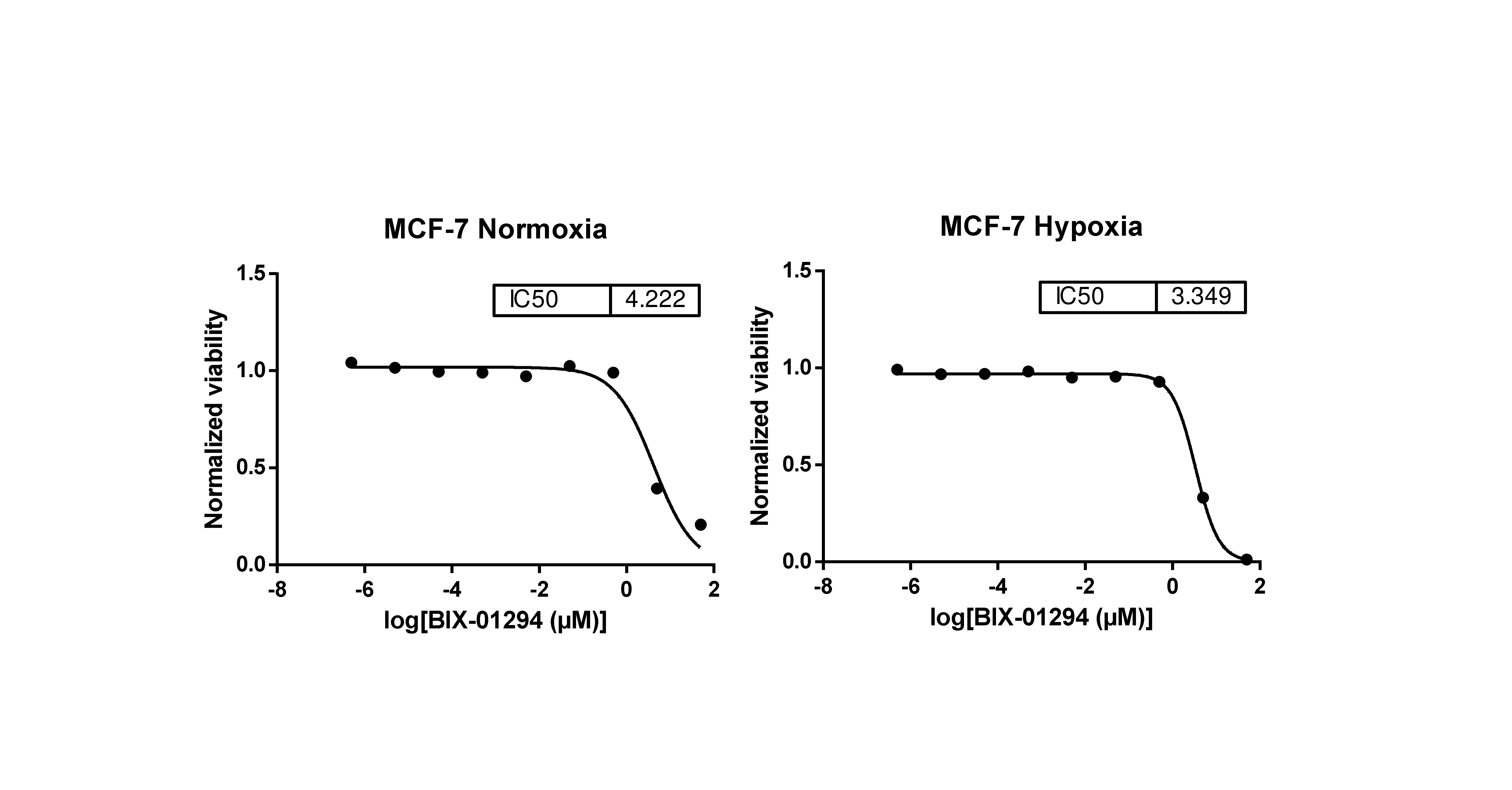

Supplement: S2 Fig — Graphs showing the normalized viability of MCF-7 cells after treatment with up to 50 μM BIX-01294 over 24 hours. The IC50 of BIX-01294 as determined by MTS was 4.222 μM in normoxia and 3.349 μM in hypoxia. Error bars indicate SEM for n = 6 replicates. (TIF) [file pone.0188051.s002.tif]

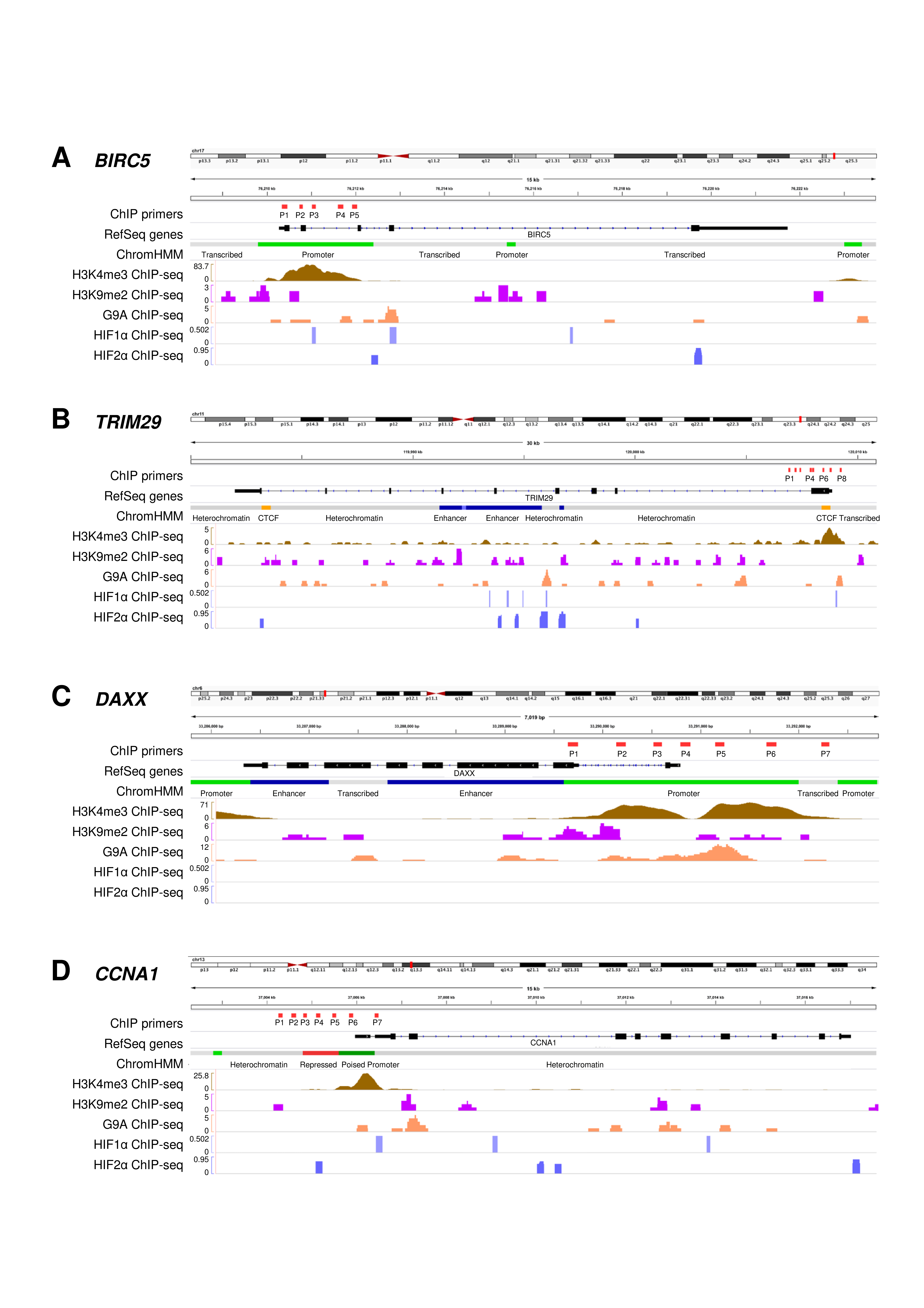

Supplement: S3 Fig — IGV profiles indicate location of primers (red rectangles), exons (black rectangles), introns (connecting black lines with blue arrows indicating direction of transcription), promoter, CTCF, enhancer and repressed regions (green, yellow, blue and red rectangles respectively), and enrichment for H3K4me3 (brown), H3K9me2 (magenta), G9A (orange) and HIF1α and HIF2α (light and dark blue respectively) for (A) BIRC5, (B) TRIM29, (C) DAXX and (D) CCNA1. (TIF) [file pone.0188051.s003.tif]

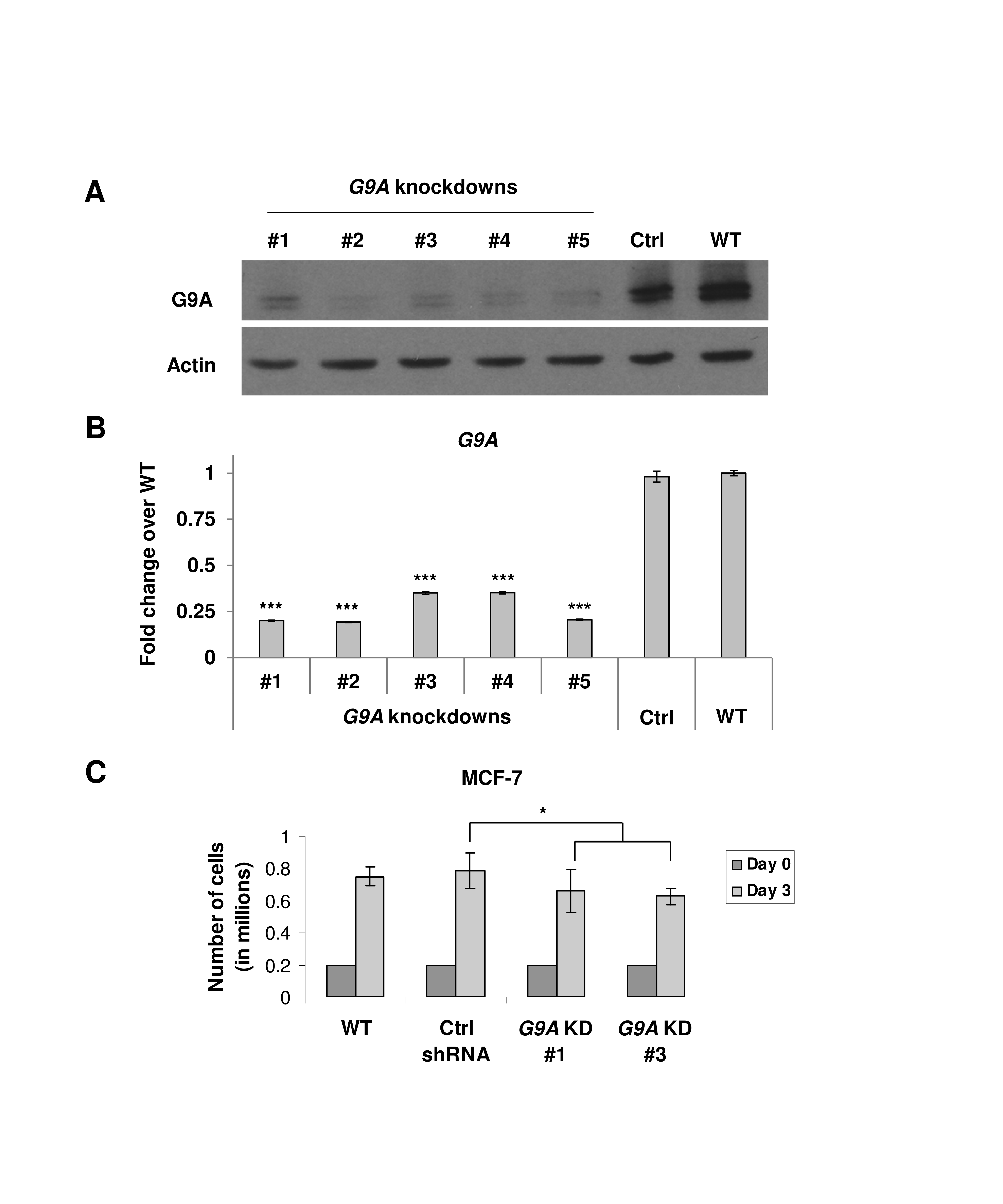

Supplement: S4 Fig — (A) Western blots showing the decrease in G9A protein levels in MCF-7 cells expressing five independent G9A shRNAs (#1 to #5) compared to the control shRNA knockdown (Ctrl) and the untreated wild-type control (WT). Actin was used as the loading control. (B) Fold change of G9A expression in five independent G9A shRNA knockdowns (#1 to #5) compared to the Ctrl and WT controls. Gene expression levels were normalized against the housekeeping reference gene EEF1G and fold change was calculated against the average of the WT controls in normoxia. Error bars indicate SEM for n = 9 replicates. (C) Bar chart showing a significantly lower number of G9A shRNA #1 and #3 knockdown MCF-7 cells after 72 hours (Day 3, light grey) from an initial seeding of 2 x 10^5^ cells (Day 0, dark grey) compared to that of the Ctrl and WT (P < 0.05). Error bars indicate SEM for n = 3 replicates. (TIF) [file pone.0188051.s004.tif]

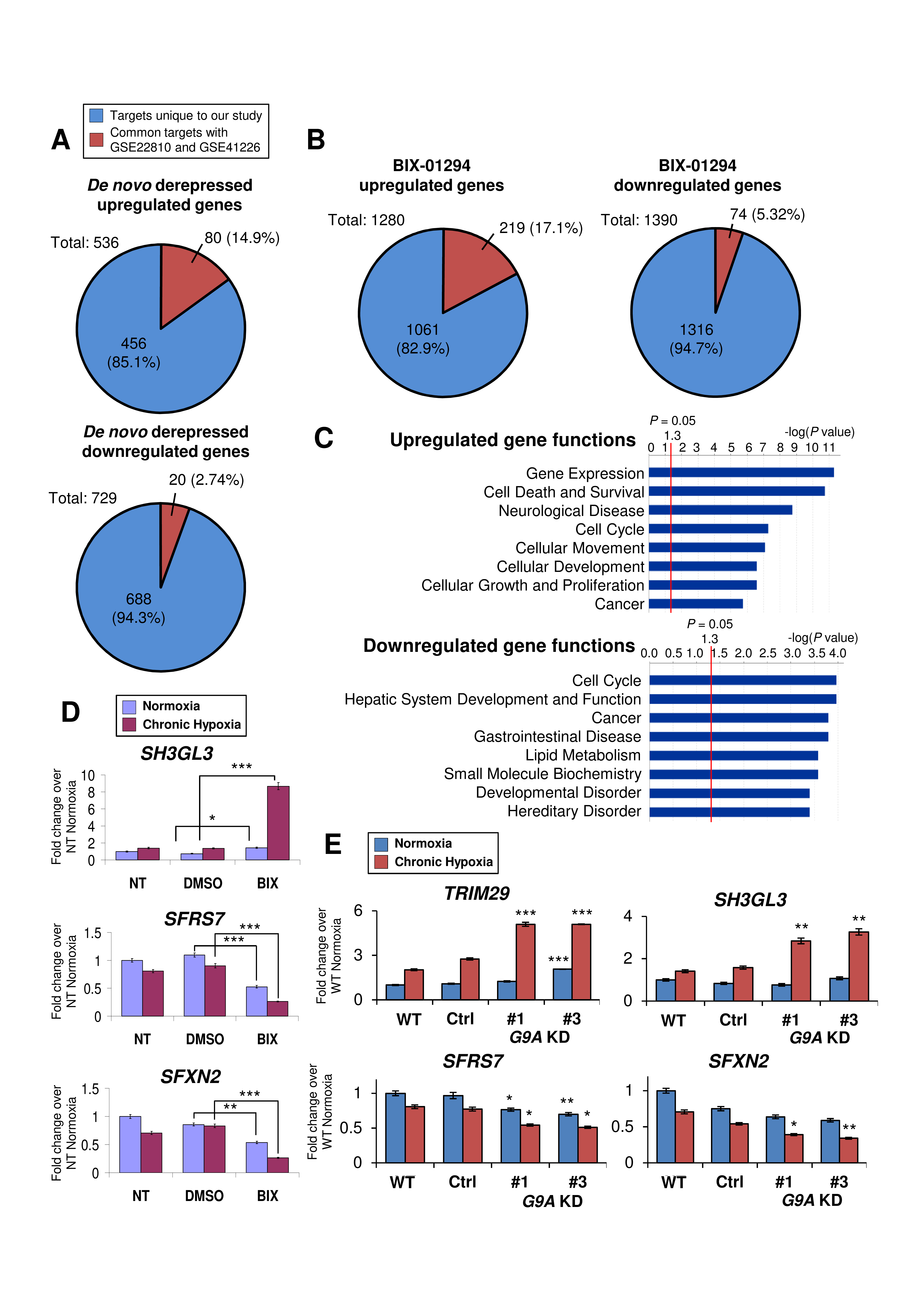

Supplement: S5 Fig — (A) Pie charts show the number of up- and downregulated de novo derepressed genes identified to also be dysregulated in the G9A microarray studies GSE22810 and GSE41226. (B) Pie charts show the number of BIX-01294 up- and downregulated genes identified to also be dysregulated in the G9A microarray studies GSE22810 and GSE41226. (C) IPA gene ontology analysis of up- and downregulated de novo derepressed genes in chronic hypoxia with BIX-01294 treatment that are differentially expressed by at least 1.5-fold over the average of the normoxic cells in BIX-01294. The top eight biological functions are shown, with a cut-off of P = 0.05 for Fisher's exact test (red lines). (D) Fold change in expression of SH3GL3, SFRS7 and SFXN2 in MCF-7 cells treated with 6 μM BIX-01294 (BIX) compared to the NT and DMSO controls in normoxia (blue) and 24 hours chronic hypoxia (magenta). Gene expression levels were normalized against the housekeeping reference gene EEF1G and fold change was calculated against the average of the NT controls in normoxia. Error bars indicate SEM for n = 9 replicates. (E) Fold change in expression of TRIM29, SH3GL3, SFRS7 and SFXN2 in MCF-7 cells expressing G9A shRNAs #1 and #3 compared to the control shRNA knockdown (Ctrl) and the untreated WT control (WT) in normoxia (blue) and 24 hours chronic hypoxia (red). Gene expression levels were normalized against the housekeeping reference gene EEF1G and fold change was calculated against the average of the WT controls in normoxia. Error bars indicate SEM for n = 9 replicates. (TIF) [file pone.0188051.s005.tif]

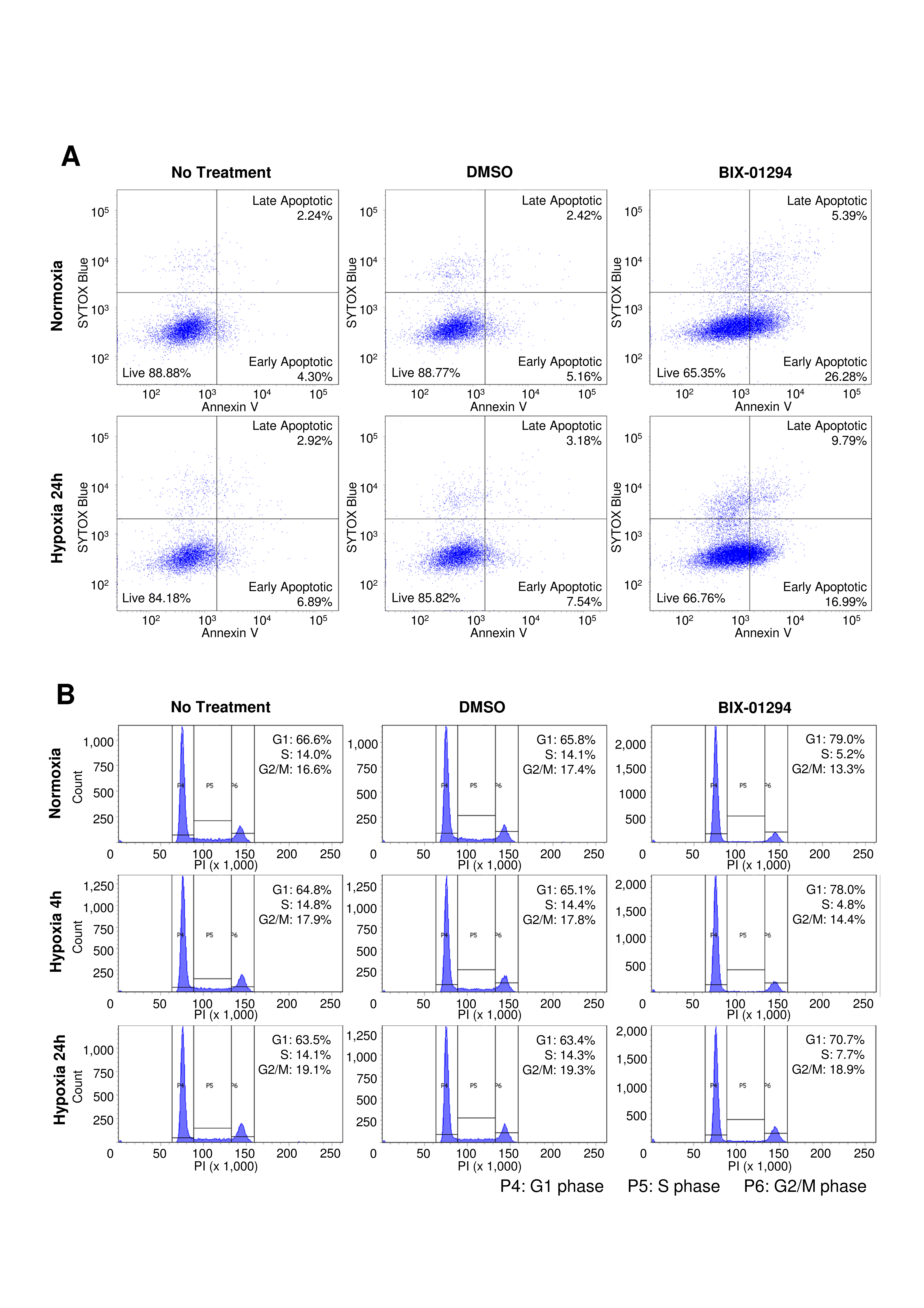

Supplement: S6 Fig — (A) Apoptosis analysis with Annexin V and SYTOX Blue stains showing the distribution of live, early apoptotic and late apoptotic MCF-7 cells treated with 6 μM BIX-01294 (BIX) compared to the no treatment and DMSO controls in normoxia and 24 hours chronic hypoxia (Hypoxia 24h). The x-axis shows fluorescence intensity from Annexin V staining indicative of cells undergoing apoptosis, while the y-axis shows SYTOX blue fluorescence, indicative of dead cells. FACS images shown are the most representative of the averages of n ≥ 6 replicates. (B) Cell cycle analysis showing the distribution of MCF-7 cells in the G1 (P4), S (P5) and G2/M (P6) phases when treated with 6 μM BIX-01294 compared to the no treatment and DMSO controls in normoxia, 4 hours acute hypoxia (Hypoxia 4h) and 24 hours chronic hypoxia (Hypoxia 24h). The x-axis shows PI fluorescence intensity, while the y-axis shows the cell count. FACS images shown are the most representative of the averages of n = 3 replicates. (TIF) [file pone.0188051.s006.tif]

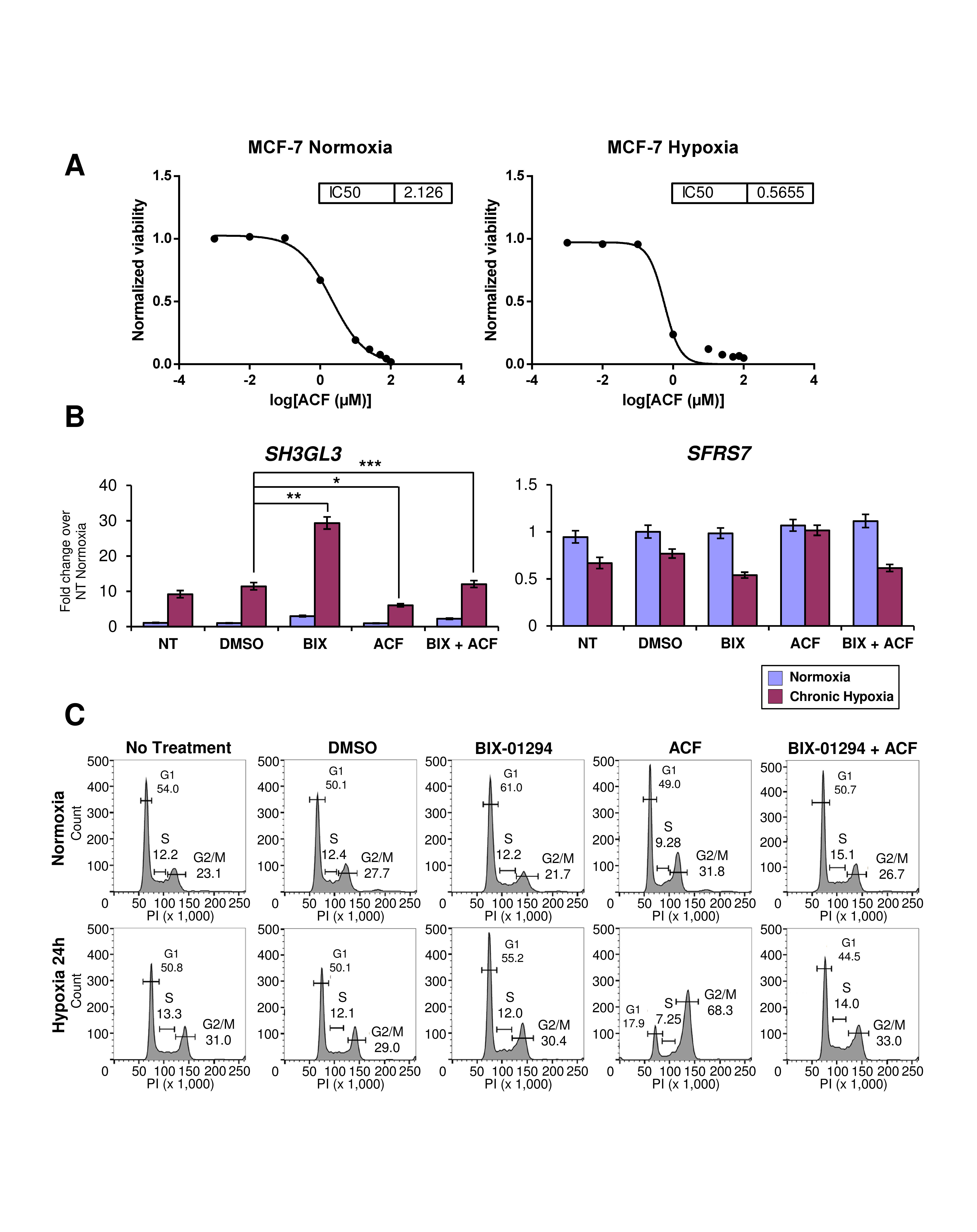

Supplement: S7 Fig — (A) Graph shows the normalized viability of MCF-7 cells after treatment with up to 100 μM ACF over 24 hours. The IC50 of ACF as determined by MTS was 2.126 μM in normoxia and 0.5655 μM in hypoxia. Error bars indicate SEM for n = 6 replicates. (B) Fold change in gene expression of SH3GL3 and SFRS7 in MCF-7 cells treated with 6 μM BIX-01294 (BIX) and/or 0.6 μM ACF compared to the NT and DMSO controls in normoxia (blue) and 24 hours chronic hypoxia (magenta). Gene expression levels were normalized against the housekeeping reference gene EEF1G and fold change was calculated against the average of the NT controls in normoxia. Error bars indicate SEM for n = 9 replicates. (C) Histograms show cell cycle analysis with the distribution of MCF7 cells in the G1, S and G2/M phases when treated with 6 μM BIX-01294 and/or 0.6 μM ACF compared to the no treatment and DMSO controls in normoxia and 24 hours chronic hypoxia (Hypoxia 24h). The percentages of cells out of the total that are in G1, S and G2/M phases are indicated. The x-axis shows PI fluorescence intensity, while the y-axis shows the cell count. FACS images shown are the most representative of the averages of n = 3 replicates. (TIF) [file pone.0188051.s007.tif]
